# Particulate Matter Exposure and Allergic Rhinitis: The Role of Plasmatic Extracellular Vesicles and Bacterial Nasal Microbiome

**DOI:** 10.3390/ijerph182010689

**Published:** 2021-10-12

**Authors:** Jacopo Mariani, Simona Iodice, Laura Cantone, Giulia Solazzo, Paolo Marraccini, Emanuele Conforti, Pallav A. Bulsara, Maria Stella Lombardi, Robert P. Howlin, Valentina Bollati, Luca Ferrari

**Affiliations:** 1 EPIGET LAB, Department of Clinical Sciences and Community Health, University of Milan, 20122 Milan, Italy; jacopo.mariani@unimi.it (J.M.); simona.iodice@unimi.it (S.I.); laura.cantone@unimi.it (L.C.); giulia.solazzo@unimi.it (G.S.); valentina.bollati@unimi.it (V.B.); 2 Department of Preventive Medicine, Fondazione IRCCS Ca’ Granda Ospedale Maggiore Policlinico, 20122 Milan, Italy; paolo.marraccini@policlinico.mi.it (P.M.); emanuele.conforti@unimi.it (E.C.); 3 GlaxoSmithKline Consumer Healthcare, Warren, NJ 07059, USA; pallav.a.bulsara@gsk.com; 4 GlaxoSmithKline Consumer Healthcare, CH-1260 Nyon, Switzerland; maria-stella.x.lombardi@gsk.com; 5 GlaxoSmithKline Consumer Healthcare, Weybridge KT13 0DE, UK; robert.x.howlin@gsk.com

**Keywords:** allergic rhinitis, particulate matter exposure, extracellular vesicles, bacterial nasal microbiome

## Abstract

Particulate matter (PM) exposure is linked to the worsening of respiratory conditions, including allergic rhinitis (AR), as it can trigger nasal and systemic inflammation. To unveil the underlying molecular mechanisms, we investigated the effects of PM exposure on the release of plasmatic extracellular vesicles (EV) and on the complex cross-talk between the host and the nasal microbiome. To this aim, we evaluated the effects of PM_10_ and PM_2.5_ exposures on both the bacteria-derived-EV portion (bEV) and the host-derived EVs (hEV), as well as on bacterial nasal microbiome (bNM) features in 26 AR patients and 24 matched healthy subjects (HS). In addition, we assessed the role exerted by the bNM as a modifier of PM effects on the complex EV signaling network in the paradigmatic context of AR. We observed that PM exposure differently affected EV release and bNM composition in HS compared to AR, thus potentially contributing to the molecular mechanisms underlying AR. The obtained results represent the first step towards the understanding of the complex signaling network linking external stimuli, bNM composition, and the immune risponse.

## 1. Introduction

Exposure to particulate matter (PM) has been linked to the worsening of several respiratory allergies [1,2,3,4,5], including allergic rhinitis (AR), which affects more than 400 million people worldwide [6]. Although the underlying mechanisms linking PM exposure and allergy remain a matter of debate [7,8,9], it is becoming increasingly clear that PM can trigger local and systemic inflammatory responses involving innate and adaptive immunity, thus potentially enhancing the exacerbation of allergic symptoms [10,11]. In this context, plasmatic extracellular vesicles (EVs), which have a critical role in the cross-talk between cells [12,13,14], are a good candidate as mediators of the airway allergic process enhancement [15,16]. Moreover, EVs strongly contribute to the modulation of the inflammatory response after PM exposure, thus enforcing this hypothesis [17,18,19]. Interestingly, EVs represent a conserved communication mechanism between domains of life; indeed they can be released by human (i.e., hEVs) and bacterial cells (i.e., bEVs) into the bloodstream [13,14,20]. Mounting evidence suggests that the bEVs released by the microbiome communities inhabiting the host mucosae play a crucial role in microbiome–host interactions also influencing the host immune response [21,22] and potentially impacting the exacerbation of allergies [23,24]. It is worthy to note that the bacterial nasal microbiome (bNM), which plays a role at the interface between the environment and the host, and has a critical role in the mediation of this interaction, inlfuences both pathogenesis and exacerbation of airway allergies, including AR [25,26,27]. 

In this context, we hypothesize that the effects of PM exposure on the EV cross-talk and the nasal microbiome composition might affect AR pathogenesis. To this aim, in the present study, we evaluate the effects of PM_10_ and PM_2.5_ exposures on both bEV and hEV release and on bNM features in 26 AR patients and 24 matched healthy subjects (HS). In addition, we assess the role exerted by the bNM as a modifier of PM effects on the complex EV-signaling network to better elucidate the systemic effects induced by PM exposure in the paradigmatic context of AR.

## 2. Materials and Methods

### 2.1. Study Population

This study included 26 adult patients with AR and 24 healthy subjects matched for age, gender, and body mass index (BMI), recruited between February and June 2019 at the Allergology Unit, Fondazione IRCCS Ca’ Granda-Ospedale Maggiore Policlinico, Milan, Italy. Each participant signed a written informed consent form, as approved by the Comitato Etico (“Milano Area 2” of the Fondazione IRCCS Ca’ Granda Ospedale Maggiore Policlinico, 20,122 Milan, Italy (approval number 157_2019bis)), following the Helsinki Declaration principles. AR was diagnosed according to the Allergic Rhinitis and its Impact on Asthma (ARIA) guidelines [28]. Inclusion criteria included subjects with AR with a positive standard battery of the skin prick test for at least one inhalant allergen and/or eosinophil count. Exclusion criteria included diabetes, hypertension, autoimmune diseases, cancer, or other major chronic health conditions (e.g., chronic and/or allergic asthma), pregnancy, and history of illicit drug use. After signing a detailed informed consent form, all participants were asked to donate blood and nasal swab samples. In addition, each participant gave his/her written informed consent to participate in the study and filled out a standardized questionnaire about demographics and lifestyle information (e.g., smoking habits and occupation). Clinical history was also collected for each of the AR patients. 

### 2.2. Particulate Matter Exposure Level Attribution

Air pollution data (PM_10_ and PM_2.5_) were retrieved from the Open Data Lombardy Region (https://www.dati.lombardia.it (accessed on 18 March 2020)) database, which contains daily estimates of municipal aggregate values calculated by the Regional Environmental Protection Agency (ARPA Lombardy). The assessment of the pollutant concentrations was based on the ARIA Regional Modelling (www.aria-net.it (accessed on 3 February 2020)), which is a chemical–physical model of air quality that simulates the dispersion and chemical reactions of atmospheric pollutants. It integrates the data measured from the monitoring stations of the ARPA Lombardy air quality network as well as meteorological data, emissions, concentrations at the beginning of the simulation period, and trends in adjacent areas, covering the whole Lombard territory with a grid of 1 × 1 km^2^ cells and providing daily mean estimates available from the website at municipality resolution. Each subject was assigned the daily PM_10_ and PM_2.5_ concentration from (1) the municipality of residence in the 6 days preceding nasal sampling (i.e., day -1 to day -6) and from (2) the Municipality of Milan (i.e., where the biological sampling was performed) for the day of recruitment (day -0).

### 2.3. 16S rRNA Gene Sequencing

#### 2.3.1. Sample Collection and Processing

Nasal swabs were collected and stored at −80 °C following standard porcedures. DNA was extracted using QIAamp^®^ UCP Pathogen Mini (Qiagen, Hilden, Germany) following the manufacturer’s guidelines. The extracted DNA was stored at −20 °C and later shipped in cold packs to the sequencing service facility Personal Genomics Srl (Verona, Italy) to perform qualitative and quantitative checks, PCR amplification, and second-generation sequencing analysis. In particular, DNA quantification was performed by the Qubit dsDNA BR assay kit (Thermofisher Scientific, Waltham, MA, USA). Libraries were generated by following Illumina 16S Metagenomic Sequencing Library Preparation (Illumina, San Diego, CA, USA). Bacterial communities were investigated through amplicon sequencing analysis of the 16S rRNA gene hypervariable regions V3-V4, amplified with the primer pair Pro341F (5′-CCTACGGGNBGCASCAG-3′) and Pro805R (5′-GACTACNVGGGTATCTAATCC-3′). The obtained libraries were evaluated by Labchip DNA High Sensitivity (Perkin Elmer, Waltham, MA, USA), quantified by the Qubit dsDNA BR assay kit (Thermofisher Scientific), and then sequenced through the Illumina MiSeq platform (Illumina) using a paired-end library of 300 bp insert size. 

#### 2.3.2. Upstream Analyses and Operational Taxonomic Unit (OTU) Clustering

Raw reads’ quality and statistics were checked using FastQC v0.11.2 and then trimmed at the 3′ end using Trimmomatic v0.32 to improve the following read joining. The Fastq-join.py tool was applied to join the raw reads and quality filtered using a minimum base quality of 20 (Phred-scale) over 5 bases’ sliding windows, and were then analyzed using the default settings for QIIME 1.9.1 [29]. After chimeric reads removal, the resulting reads were clustered using 97% similarity, applying the open-reference OTU pipeline using USEARCH61 [30]. Taxonomic assignment was carried out with the RDP classifier [31] through the comparison of representative reads against the Greengenes v13.8 database using standard options. The PyNast method and default settings suggested in the QIIME pipeline were applied to align sequences [32,33]. The resulting OTU table was successively filtered, removing singleton and low abundance OTUs (i.e., <1% relative abundance) to perform downstream and statistical analysis. The final OTU table contained 1,941,731 reads (sequences length (mean ± standard deviation [SD]) = 408 bp ± 37 bp).

#### 2.3.3. Downstream Analysis

Downstream analyses were carried out using QIIME v1.9.1 to analyze the above-described OTU table. Taxonomic values within each sample and group were assigned to each OTU from the phylum to the genus level. OTUs, which failed genus attribution, were tagged as “Unassigned” followed by the specific family label. Before diversity analysis, all samples were rarefied to 10,000 sequences with a seed of 10 to avoid the influence of different sequencing depths, as this number of sequences was the minimum identified in the OTU table. Alpha-diversity richness, evenness, and genetic distance were calculated using observed species, Shannon, PD whole tree, Chao 1, and Simpson indices. Beta-diversity was examined by applying the weighted normalized UniFrac distance measure. 

### 2.4. EVs Analysis

The isolation, purification, and characterization of EVs were performed by following MISEV 2018 guidelines [34].

#### 2.4.1. Isolation and Purification of EVs

Blood samples were collected in ethylenediamine tetra-acetic acid (EDTA) tubes and processed within 2 h from the phlebotomy as described below. For isolation of plasma EVs, two aliquots of 3 mL of plasma for each subject were subsequently centrifuged at 1000, 2000, and 3000× *g* for 15 min at 4 °C. The obtained pellets were discarded to remove cell debris. EVs were then isolated from supernatants by ultracentrifugation at 110,000× *g* for 94 min at 4 °C in polypropylene ultracentrifuge tubes (Beckman Coulter; Brea, CA, USA) filled with phosphate-buffered saline (PBS) previously filtered through a 0.10 μm pore-size polyethersulfone filter (StericupRVP, Merck Millipore; Burlington, MA, USA). To carry out the Nanoparticle tracking and flow cytometry analyses, the EV-rich pellet was resuspended in 1 mL of triple-filtered PBS (pore size 0.1 µm). The methods described here are further detailed in the supplementary information (Supplementary Table S1).

#### 2.4.2. Nanoparticle Tracking Analysis (NTA) of EVs

NTA analysis was carried out with the Malvern NanoSight NS300 system (Malvern Panalytical Ltd., Malvern, UK) used to visualize the EVs by laser light scattering. For each sample, five 30 s records were registered. NTA output was then analyzed with integrated NTA software (Malvern Panalytical Ltd.), providing high-resolution particle size distribution profiles and EV concentration measurements. NTA EV data were expressed as 10^6^ for 1 mL of plasma.

#### 2.4.3. Flow Cytometry on EVs

To determine EV cellular origins, immunophenotyping was achieved with the MACSQuant Analyser flow cytometer (Miltenyi Biotec, Bergisch Gladbach, Germany, https://bit.ly/3BuB6ze (accessed on 8 October 2021), The Fluoresbrite Carboxylate Size Range Kit I (0.2, 0.5, 0.75, and 1 µm) was used to set the calibration gate on the MACSQuant Analyser system. To evaluate the integrity and to highlight the hEV subset, 60 µL sample aliquots were stained with 0.02 µM 5(6)-carboxyfluorescein diacetate N-succinimidyl ester (CFSE) at 37 °C for 20 min in the dark. CFSE is a vital dye non-fluorescent molecule able to enter into EVs, where intracellular esterase enzymes remove the acetate group and convert the molecule into the fluorescent ester form. To characterize and count hEVs, the following panel of antibodies was used: AbCD177-APC (neutrophils, clone REA 598; APC: allophycocyanin, Miltenyi Biotec), AbCD14-APC (monocytes, clone TUK4, Miltenyi Biotec), AbCD61-APC (platelets, clone Y2/51, Miltenyi Biotec), CD326 (EpCAM)-APC (epithelium, clone HEA-125, Miltenyi Biotec), AbCD62E-APC (activated epithelium, clone REA280, Miltenyi Biotec), AbCD203C (mast cells, clone REA 826, Miltenyi Biotec), and AbCD294 (eosinophils, clone REA 598, Miltenyi Biotec). In addition, two different 60 µL sample aliquots for each sample were also incubated with specific antibodies to recognize the bEVs: Lipopolysaccharide (LPS; gram-negative; Invitrogen, Waltham, MA, USA) and a primary unconjugated Lipoteichoic acid (LTA; gram-positive; Invitrogen) combined with a secondary IgG-antimouse (IgG (H + L), Superclonal™ Recombinant Secondary Antibody, Alexa Fluor 647-Invitrogen) to distinguish EVs derived from gram-negative or gram-positive bacteria from the whole subset. Before use, each antibody was centrifuged at 17,000× *g* for 30 min at 4 °C to eliminate aggregates. A stained PBS blank sample was used to detect the auto-fluorescence of the antibody. Quantitative multiparameter analysis of flow cytometry data (expressed as 10^3^ for 1 mL of plasma) was run using FlowJo software (Tree Star, Inc., Ashland, OR, USA). Antibody gating strategies are reported in the supplementary information (Supplementary Figures S1–S4) and were previously described [17,35].

### 2.5. Statistical Analysis

Standard descriptive statistics were performed on all variables. Categorical data are presented as frequencies and percentages. Continuous variables are expressed as the mean ± standard deviation (SD). 

The differences between the two groups of AR and HS subjects were evaluated through Pearson’s chi-square test or Fisher’s exact test for categorical data, or *t*-test or the Mann–Whitney U-test for continuous variables, as appropriate. 

To evaluate the association between PM_10_ and PM_2.5_ exposure on the different EVs subtypes, in AR and HS, we applied negative binomial regression for over-dispersed count observations. We tested the presence of over-dispersion based upon the Lagrange Multiplier (LM) test. Under the hypothesis that a short-term mechanism underlies variations in EV levels, we chose to investigate the exposure at the day of recruitment (defined as day -0). Models were adjusted for age, gender, smoking habit, mean temperature, mean humidity, case-control condition, and interactions between case-control and PM exposure. 

Each model was tested for normality and linearity. All potential confounders were included in the multivariate model after verifying the presence of an association in a univariate model. Best model selection was based on the minimization of the Akaike information criterion and the maximization of the explained variance of the model. Estimated effects are reported as β and 95% confidence intervals (CI) associated with an increase of 10 units in each pollutant. To verify the feasibility of stratifying the analyses for allergy status (AR and HS), we tested the interaction between allergy status and exposure.

We analyzed the differences in AR and HS in terms of the distribution of vesicle marginal mean concentrations for each EV size. For each EV size: (1) we estimated marginal EVs’ mean concentration and 95% CI at each size, with multivariate negative binomial regression models adjusted for age, gender, smoking habit, mean temperature, mean humidity, case-control condition, PM_10 (day -0)_, and interactions between case-control and PM exposure; (2) we compared the EV mean differences between AR and HS; and (3) we calculated q-FDR values using the multiple comparison methods based on Benjamini–Hochberg False Discovery Rate (FDR) that takes into account the high number of comparisons, with a threshold of 0.10 to detect significance. Results were reported as a series graph for EV mean concentrations of each size and vertical bar charts to represent the size-specific *p*-values and *q*-values (i.e., FDR *p*-values) obtained comparing AR and HS. In the graphs, the *x*-axis corresponds to the size of EVs.

To assess differences between the OTU abundances and the alpha-diversity indices in the AR and HS groups, the *t*-test was applied.

Beta-diversity was examined by applying the weighted normalized UniFrac distance measure. To compare the tightness of the clustering distance within all the samples in the two groups comparing the state of disease, we performed a non-parametric permutational multivariate analysis of variance (NPMANOVA). This is used to compare groups of subjects and tests the null hypothesis that the centroids and dispersion of the groups, as defined by measure space, are equivalent for all groups. The test is based on the prior calculation of the distance between any two subjects and the rejection of the null hypothesis means that the centroid of the subject is different between the groups. Furthermore, to visualize and interpret the result of the applied distance measure, principal coordinate analysis (PCoA) was performed on the produced distance matrix and plotted using Emperor, and the adonis function in the R Vegan package was used to test the significance in dissimilarity matrices between AR and HS groups.

To evaluate the effects of PM_10_ and PM_2.5_ on α-diversity indices, for both the AR and HS groups, we applied multivariable linear regression models, adjusting for age, gender, smoking habit, mean temperature, mean humidity, allergy status, and interactions between allergy status and PM exposure. The association between PM and α-diversity indices was evaluated from the sixth (day -6) to the day of sampling (day -0), running several regression equations separately but reporting those on day -5, which were the only statistically significant associations we observed. Therefore, we hypothesized that day -5 of PM exposure might influence bNM composition and this, in turn, might affect the plasmatic EV release in response to acute PM exposure (i.e., day -0).

Factor analysis was applied to identify a small number of underlying independent variables and microbiomes factors that explain most of the variance that was observed in the much larger number of manifest variables of the microbiome composition at the genus level.

We excluded a priori 16 genera (i.e., “unassigned”, and “other”) because they did not provide any interpretable results and 21 genera with a very low relative abundance (mean ≤4% and median ≤0.3%). Next, we analyzed the correlation matrix of the log-transformed and standardized variables to investigate how genera correlated to each other and we took into account existing relationships among genera to avoid over-representation of single genera and the subsequent artificially higher correlations. Since three genera did not correlate (*p*-value < 0.05) with any other genera and correlation coefficients were less than |0.30|, they were not included in the factor analysis. We evaluated whether the correlation matrix of the relative abundances of the genera was factorable through both visual inspections of the matrix and statistical procedures, including Bartlett’s test of sphericity, the Kaiser–Meyer–Olkin measure, and individual measures of sampling adequacy. Bartlett’s test of sphericity tests the hypothesis that the correlation matrix is an identity matrix, which would indicate that genera variables are unrelated and therefore not suitable for structure detection. A significant *p*-value indicates that factor analysis may be useful with our data. The Kaiser–Meyer–Olkin measure of sampling adequacy indicates the proportion of variance in the genera variables that might be caused by underlying factors. The overall and individual measure of sampling adequacy ranges between 0 and 1. We considered an overall KMO ≤ 0.50 for the factor analysis and genera with a measure of sampling adequacy <0.30 as unacceptable. Thus, we excluded 17 genera and again verified the method assumption on the correlation matrix considering the remaining 24 genera. The new correlation matrix was factorable but five genera were excluded because of their low communality, i.e., they explained less than 15% of the variance each. In the last correlation matrix, all the assumptions were satisfied and brought the results obtained (Supplementary Table S2).

We carried out an explanatory factor analysis on the correlation matrix of 19 selected microbiome genera to derive a smaller set of uncorrelated underlying factors and to obtain the microbiome patterns (Supplementary Table S3).

We chose two factors to be included in the analysis considering the following criteria: factor eigenvalues >1, scree-plot construction, and factor interpretability [36]. We applied a varimax rotation to the factor-loading matrix to obtain a simpler loadings structure and make the factor matrix more interpretable. We calculated the factor scores using the mean principal components with iteration. Genera with an absolute rotated factor loading ≥0.63 on a given factor were used to name the factor and are indicated as “dominant genera” hereafter [37]. The factor scores, calculated for each subject, indicate the degree to which each subject’s microbiome corresponded to the two identified patterns. To assess the reliability of microbiome patterns and internal consistency of genera that load more than |0.40| on any factor, we calculated Cronbach’s coefficient alpha for each factor and coefficient alpha when the item was deleted.

We investigated if the two microbiome patterns identified by factor analysis could act as a modifier of the association between PM exposure and the different EVs subtypes. Thus, we investigated the role of microbiome patterns in the relationship between PM exposure and different EVs subtypes, applying multiple negative binomial regression models adjusted for age, gender, smoking habit, mean temperature, mean humidity, allergy status, PM_10 (day -0)_, and the interactions between microbiome factors (Factor1 or Factor2, evaluated in separate models) and PM exposure. We reported the association between PM and different EVs subtypes adapted for the selected values of each factor (i.e., the 5th quantile, median, and the 95th quantile) and the other covariates.

All statistical analyses were performed by using SAS 9.4 statistical software (SAS Institute Inc., Cary, NC, USA) and R version 4.0.2.(R Core Team, Vienna, Austria)

## 3. Results

### 3.1. Study Population and PM Exposure Data

The study population included 50 subjects (26 AR patients and 24 HS). No differences were found between AR (i.e., patients with allergic rhinitis) and HS (i.e., healthy matched controls) groups in any of the socio-demographic characteristics factors (Table 1). Patients’ mean age was 41 years (±14.6 years) and the mean BMI was 23.8 kg/m^2^. Age, BMI, and gender values were matched in the two study groups. In total, 38% of enrolled subjects were smokers. Median PM_10_ and PM_2.5_ values in the week before the enrolment were 22.9 µg/m^3^ and 14.9 µg/m^3^, respectively. A description of the PM_10_ and PM_2.5_ fractions for each considered day (from day -0 to day -6) is reported in Supplementary Figure S5.

### 3.2. EV Characterization in the AR and HS Groups

The median total number of EVs between AR patients and HS (i.e., total EVs within Table 2) was investigated by NTA analysis and AR patients were characterized by a higher concentration of plasmatic EVs than HS (EV median in AR: 1267 × 10^6^/mL vs. EV median in HS: 9688 × 10^6^/mL; *p* = 0.048).

Moreover, we characterized plasmatic EVs, focusing on both bEVs and hEVs subpopulations by flow cytometry analysis. Consistent with the NTA results, the median concentrations of all EVs subtypes were higher in AR patients compared to those of the HS group (Table 2). The median concentrations of bEVs released from eosinophils (CD294 + EVs) and basophils (CD203C + EVs) were significantly increased in AR patients compared to the HS group.

### 3.3. Association between PM Exposure and Plasmatic EV Concentrations in AR and HS Group

We investigated the association between PM_10_ and PM_2.5_ exposure and both bEV and hEV plasmatic concentrations in the analyzed groups (Table 3). Considering the HS group, we observed a negative association between PM_10 (day -0)_ levels and the plasmatic concentration of LTA + EVs (β = −0.38, *p* = 0.013), LPS + EVs (β = −0.38, *p* = 0.023), and CD14 + EVs (β = −0.44, *p* = 0.013). No associations were found for AR patients.

**Figure 1:**
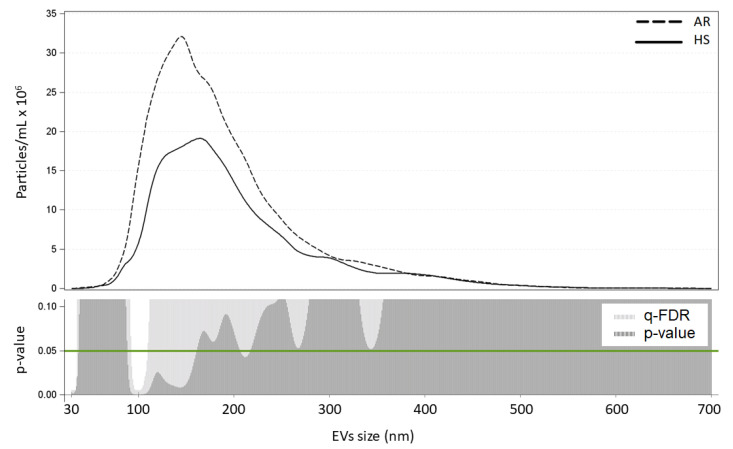
Number of EVs for each size (nm) in HS and AR. The upper panel of the figure reports EV concentration for each size for the AR (dashed line) and HS (solid line) groups. The lower panel reports the *p* and *q*-values of the comparisons of each size EV for the entire 30–700 nm size range (AR vs. HS). EV concentrations were calculated as marginal means from a negative binomial model adjusted for age, gender, smoking habit, mean temperature, mean humidity, case-control condition, PM_10 day -0_, and interactions between case-control and PM exposure.

We further compared the AR patients and HS in terms of the distribution of marginal mean vesicle concentrations for each size (Figure 1). AR patients were characterized by a higher concentration of EVs ranging between 30 and 35 nm, 92–111 nm (*p* < 0.05; *q* < 0.10), with a peak at 99 nm.

### 3.4. Compositional Overview of the Nasal Microbiota between AR and HS Participants

**Figure 2:**
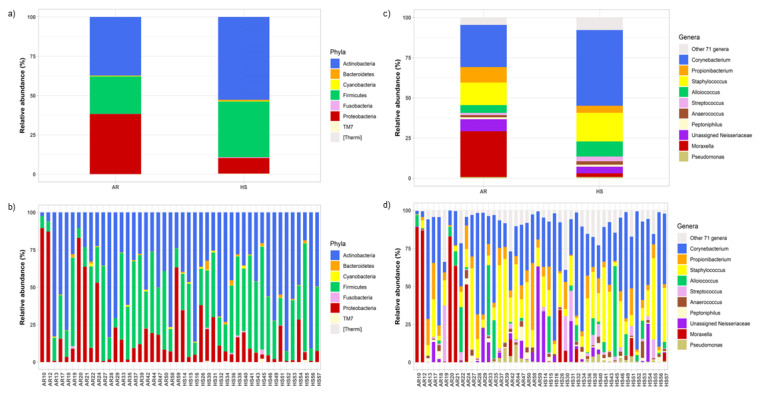
bNM phylum and genus-profile composition. (**a**) Average relative abundance of the bNM structure in the AR and HS groups; (**b**) relative abundance of the bNM of each enrolled subject; (**c**) average relative abundance in the AR and HS group; and (**d**) genus-profile structure of the bNM for each recruited subject. In addition, α-diversity analyses were performed to investigate compositional species richness and evenness within the study groups. The AR group was characterized by less diverse bNM compared to the HS group, as the bNM of the AR patients expressed a reduced mean richness and phylogenetic diversity compared to the HS group, as shown by the observed species (*p* = 0.009), Chao1 (*p* = 0.023), and the PD whole tree (*p* = 0.009) indices (Table 4).

A sufficient yield for amplicon-based analysis was obtained from 23 AR patients and 23 HS samples. Considering the entire study population as a whole, the bNM was dominated by the Actinobacteria (abundance range AR: 2.5–83.5%; abundance range HS: 18.6–92.7%), Firmicutes (AR: 6.1–63.5%; HS: 6.4–72.2%), and Proteobacteria (AR: 0.6–89.6%; HS 0.7–38%) phyla. We compared the mean relative abundance of each phylum in the two groups and observed that the Proteobacteria phylum (mostly represented by Actinobacteria and the Firmicutes) was higher in the AR group compared to HS, although this evidence did not reach the significance level (Figure 2a). Interestingly, among the AR patients, six subjects were characterized by a very high presence of the Proteobacteria phylum. The remaining 17 AR patients seemed to exhibit a more heterogeneous composition with high relative abundances for the Actinobacteria and the Firmicutes phyla (Figure 2b). No differences were observed in terms of clinical, demographic, and lifestyle information between the six AR patients with a high prevalence of Proteobacteria phylum and the remaining 17 AR patients (data not shown).

We identified 81 genera represented in the study population. The most represented genera were Corynebacterium (AR: 2.3–71.3%; HS: 6.9–91.0%), Staphylococcus (AR: 0.2–55.0%; HS: 1.7–53.6%), Moraxella (AR: 0.006–88.9%; HS: 0–34.4%), Propionibacterium (AR: 0.14–44.1%; HS: 0.73–17.9%), Alloiococcus (AR: 0.005–46.2%; HS: 0.015–59.6%), Unassigned_Neisseriaceae (AR: 0.003–62.8%; HS: 0–33.4%), Streptococcus (AR: 0.005–37%; HS: 0.1–22.6%), Anaerococcus (AR: 0.01–5.5%; HS: 0.006–10.9%), Peptoniphilus (AR: 0.001–9.24%; HS: 0.005–4.9%), and Pseudomonas (AR: 0.002–11.6%; HS: 0.006–5.4%). Stratifying by the AR and HS group, the AR group showed a tendency of increased relative abundance for the Moraxella genus (belonging to Proteobacteria phylum), while Corynebacterium and Staphylococcus genera abundances were higher in the HS group (Figure 2c,d).

Distances between groups of subjects were additionally analyzed through the weighted normalized UniFrac distance metric. Intra-group distance distribution was significantly greater in the AR patients than in the HS (non-parametric test = 3.77, *p* = 0.01; Supplementary Figure S6a). To visualize this intra-group variability, a PCoA plot was generated (Supplementary Figure S6b). The first two axes, PC1 and PC2, were found to explain 66.9% of the bNM variability across the study population. The PCoA confirmed the greater dispersion of the AR subjects compared to the HS. However, no differences between the considered groups were identified (R^2^ = 0.045 *p* = 0.10).

### 3.5. Exploratory Factor Analysis

An exploratory factor analysis was conducted to determine which latent variables (i.e., factors) modify the relationship between PM exposure and EV release. Factor analysis was applied to identify a small number of underlying independent variables, namely microbiomes factors, that explain most of the variance that is observed in the much larger number of manifest variables of the microbiome composition at the genus level.

**Figure 3:**
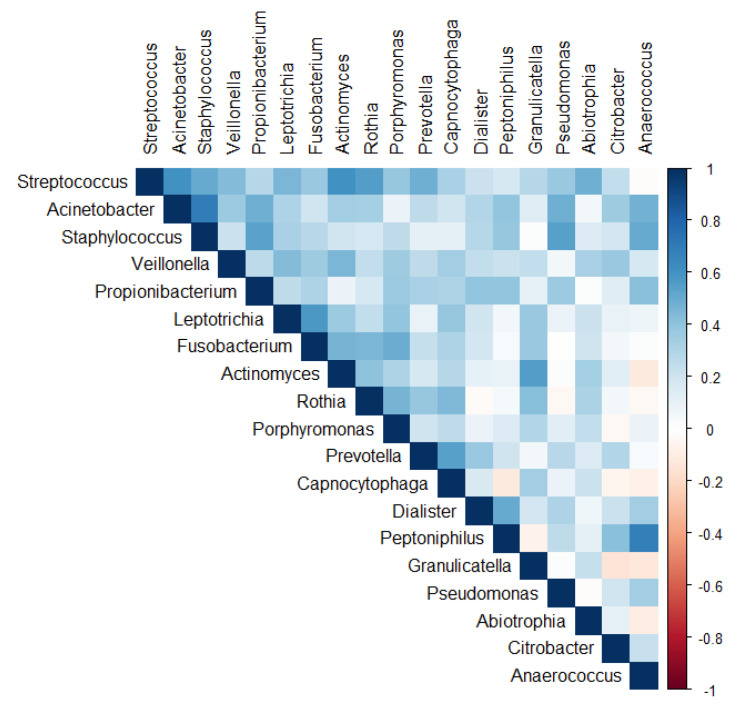
Correlation matrix of the 19 genera used in the factor analysis.

The correlation matrix of the 19 selected genera (Figure 3) was suitable for factor analysis. Supplementary Table S3 reports the results on statistical procedures for checking matrix factorability. Bartlett’s test of sphericity was significant (*p* < 0.001). The overall measure of the sampling adequacy was equal to 0.62, indicating that the sample size was sufficient, as compared to the number of genera under consideration. In addition, the individual measures of the sampling adequacy were satisfactory. Table 5 shows the factor-loading matrix for the two retained microbiome patterns, namely the corresponding communality estimates and the proportion of explained variance. The two retained factors explained about 66% of the total variance of the 19 genera, accounting for about 44% and 22%, respectively. The greater the loading of a given genus to one factor, the higher the contribution of that genera to the factor. The first factor, named Factor1, had the highest contribution from *Streptococcus, Actinomyces, Rothia,* and *Fusobacterium*. The second factor, named Factor2, was characterized by the greatest positive loadings on *Anaerococcus, Acinetobacter, Peptoniphilus,* and *Staphylococcus*. All the examined genera had at least one-factor loading greater than |0.39|, thus proving an important role of all the genera included in the analysis.

### 3.6. PM Effects on bNM Composition

We evaluated the effects of PM_10_ and PM_2.5_ exposure on the α-diversity (from day -6 to day -0) for both the AR and HS groups. As shown in Table 6, in the AR subjects, positive associations were identified only between the fifth day preceding sampling PM_2.5 (Day-5)_ levels and observed species (PM_2.5_ β = −34.2, *p* = 0.01), the PD whole tree (PM_2.5_ β = 1.8, *p* = 0.036), and Shannon (PM_2.5_ β = 1.2, *p* = 0.04) indices. On the contrary, negative associations were observed in HS among PM_2.5_ and observed OTUs (PM_2.5_ β = −22.5, *p* = 0.032), Shannon (PM_2.5_ β = −0.97, *p* = 0.041), Chao1 (PM_2.5_ β = −25.3, *p* = 0.097), and Simpson (PM_2.5_ β = −0.2, *p* = 0.012) diversity indexes. No significant associations were observed with PM_10_ exposure.

### 3.7. Association between PM Exposure and Plasmatic EV Concentration in the Whole Population, Stratified According to Factors

We investigated the role of bNM in terms of microbiome patterns as a possible modifier effect of plasmatic EV release after PM exposure at day -0. The group with a bNM dominated by high levels of Factor1 (including *Streptococcus, Actinomyces, Rothia,* and *Fusobacterium*) showed negative associations with LTA+, LPS+, and CD14 + EVs when considering PM_10 (day -0)_ exposure. For PM_2.5_, we observed a negative association between high levels of Factor1 and CD14 + EVs (Table 7). The group with a bNM dominated by high levels of Factor2 (including *Anaerococcus, Acinetobacter, Peptoniphilus,* and *Staphylococcus*) showed negative associations with LPS + when considering PM_10 (day -0)_ exposure No associations were observed for PM_2.5_ (Table 8).

## 4. Discussion

The prevalence of respiratory diseases is rising dramatically worldwide, turning respiratory allergies into an “epidemic” phenomenon [37,38,39]. In particular, severe AR has been associated with significant impairments in quality of life, work performance, and sleep [39,40]. Increasing epidemiological studies conducted both on adults and children have been reporting an associative relationship between air pollution, including PM, and increased incidence of AR [7,9,41,42,43,44,45,46]. Indeed, allergens and environmental agents trigger reactive nasal inflammatory conditions such as AR via the involvement of host intrinsic factors, including the innate and adaptive immune system, as well as the bNM membership [47,48]. In order to highlight the molecular mechanisms linking air pollution exposure and AR, we investigated both plasmatic EVs and bNM as potential actors in this complex scenario.

We assessed plasmatic EV release in AR compared to HS and the higher plasmatic EV concentrations found for the AR group could be linked to pro-inflammatory activation, as it was previously reported [49,50,51]. Regarding the hEVs fraction, the increased concentrations of both mast cells and neutrophils-derived EVs may denote the enhanced IgE-based immune response, a specific signature of allergic conditions [52,53]. We observed a tendency of increased concentration also for monocyte and platelet-derived EVs, which release was reported to be increased during proinflammatory stimuli, also mediated by environmental exposure [54,55,56]. When considering the PM effect, the AR and HS groups showed an opposite tendency between day -0 PM_10_ exposure and EVs released by both gram-positive and gram-negative bacteria (i.e., LTA+ and LPS + EVs, respectively), although only the negative associations in HS reached the statistical significance level. This evidence suggests that PM exposure influences bNM cross-talk with the host, affecting bEV release [57]. In particular, in-vitro studies emphasized that bEVs can trigger inflammation through the activation of the toll-like receptors 2 and 4 [58,59]. 

The complex interaction between the microbiome and the host is at the basis of human health. Microbiome dysbiosis can be caused by exposure to several environmental pollutants, including PM, and has been associated with numerous diseases. Nonetheless, the role of the microbiome in the multifaceted host–environment interplay is far from being fully understood [21]. Both HS and AR bNMs were dominated by the Actinobacteria, Proteobacteria, and Firmicutes phyla according to previous studies [21]. Interestingly, the high Proteobacteria-relative abundance, which characterized the bNM of the AR patients, has been linked to several respiratory diseases, such as chronic obstructive pulmonary disease (COPD), tuberculosis, and asthma [60,61,62]. 

The importance of a diverse microbiome has been widely documented regarding maintaining mucosa integrity and an effective immune system [21,63]. Thus, the less diverse bacterial community observed in the AR group compared to the HS group may be linked to the disease condition. In particular, reduced diversity in the bNM was identified also in other respiratory diseases such as chronic rhinosinusitis [23,64], asthma [65], and granulomatosis with polyangiitis [66]. This evidence is further supported by the opposite behavior observed between PM_2.5_ and α-diversity indices in HS and AR, possibly leading to an imbalanced or dysfunctional microbial community with an impact on disease severity and inflammatory response [21,57,67,68,69,70,71]. 

Since bNM is a complex and dynamic community [72], we applied factor analysis to obtain a more comprehensive picture of the most representative bNM composition in the study group, thus considering a smaller number of independent factors able to predict microbiome composition at the genus level. The factorial analysis allowed us to further investigate whether bNM is able to modify the effects induced by PM exposure on both bEVs and hEVs concentrations. To this aim, we considered the two factors (i.e., Factor1 and Factor2) identified, tested the interaction between the two categories and the PM exposure in the multivariable regression model, and observed different associations depending on the factor. This evidence supports our hypothesis that bNM composition is influenced by PM and, in turn, this effect modifies the acute plasmatic EV release after PM exposure. In accordance with this, we recently reported that in healthy subjects, bNM membership was linked to the pattern of plasmatic EV release when PM was considered [73].

We need to acknowledge that the study has some limitations. The present observational study was conducted by taking into account a size-limited population, which, consequently, may not be sufficient to uncover all the variations induced by the PM effect, especially when either EV concentration or the bNM feature were considered. Moreover, we estimated PM exposures at the participants’ residential addresses, without considering their daily activities and locations, nor the indoor PM levels, which may have resulted in misclassifications of the exposure attributions. However, the consistency of our findings with the literature makes us confident that the obtained results were not found by chance and help to understand the complex signaling network, thus linking external stimuli, bNM composition, and the immune system.

## 5. Conclusions

In the present study, we observed an association between PM exposure in the context of both bNM composition and plasmatic EV release in patients with AR. Moreover, we observed that PM exposure differently influences plasmatic EV release depending on the bNM composition. To the best of our knowledge, this is the first study specifically investigating this association. Longitudinal studies will help to clarify the link between PM exposure and both bNM modulation and plasmatic EV release in those subjects with the AR condition. Moreover, further functional studies are needed to better characterize the different responses observed in the AR and HS groups after PM exposure, as well as to investigate the specific cell subpopulations involved in this mechanism and the consequent gene expression variations.

## Figures and Tables

**Table 1:** Characteristics of the study participants (N = 50).

Characteristics	HS Controls (*n* = 24)	AR Cases (*n* = 26)	All Subjects (*n* = 50)	*p*-Value
Age, years, mean ± SD	42.7 ± 16.4	39.4 ± 12.8	41.0 ± 14.6	0.439
Gender, *n* (%)				
*Males*	13 (54%)	13 (50%)	26 (52%)	0.768
*Females*	11 (46%)	13 (50%)	24 (48%)	
Smoking, *n* (%)				
*Yes*	9 (37%)	10 (38%)	19 (38%)	0.944
*No*	15 (63%)	16 (62%)	31 (62%)	
BMI, kg/m^2^, mean ± SD	23.8 ± 3.7	23.7 ± 3.1	23.8 ± 3.3	0.898
Residential area, *n* (%)				
*City*	12 (50%)	15 (58%)	27 (54%)	0.145
*Small town*	8 (33%)	3 (11%)	11 (22%)	
*Suburbs*	4 (17%)	8 (31%)	12 (24%)	
Education, *n* (%)				
*Less than high school*	4 (17%)	1 (4%)	5 (10%)	0.182
*High school or university*	20 (83%)	25 (96%)	45 (90%)	
Children, *n* (%)				
*Yes*	12 (50%)	14 (54%)	26 (52%)	0.908
*No*	11 (46%)	12 (46%)	23 (46%)	
Occupation, *n* (%)				
*Employed*	21 (88%)	24 (92%)	45 (90%)	0.571
*Unemployed/retired*	3 (12%)	2 (8%)	5 (5%)	
Usage of disinfectants, *n* (%)	11 (46%)	15 (58%)	26 (52%)	0.402
*Surface cleaning*	7 (29%)	14 (54%)	21 (42%)	0.077
*Clothing cleaning*	4 (17%)	8 (31%)	12 (24%)	0.243
*Fruit and vegetable cleaning*	3 (13%)	0 (0%)	3 (6%)	0.103
Frequency of use, *n* (%)				
*Never*	13 (54%)	10 (39%)	23 (46%)	0.408
*Rarely*	3 (13%)	4 (15%)	7 (14%)	
*Often*	1 (4%)	5 (19%)	6 (12%)	
*Habitually*	7 (29%)	7 (27%)	14 (28%)	
Pets, *n* (%)	7 (29%)	6 (23%)	13 (26%)	0.624
Dog	2 (8%)	5 (19%)	7 (14%)	0.225
Cat	4 (17%)	1 (4%)	5 (10%)	
Rabbit	1 (4%)	0 (0%)	1 (2%)	
Allergen				
*Grass pollens*	0 (0%)	17 (65%)	17 (34%)	<0.001
*Compositae*	2 (8%)	11 (42%)	13 (26%)	0.006
*Cat/dog*	0 (0%)	3 (12%)	3 (6%)	0.236
*Moulds*	0 (0%)	5 (19%)	5 (10%)	0.051
*Birch pollen*	0 (0%)	14 (54%)	14 (28%)	<0.001
*House dust mite*	1 (4%)	13 (50%)	14 (28%)	<0.001
Leukocyte formula, 10^9^/L, mean ± SD				
*Neutrophils*		5.6 ± 6.9		
*Lymphocytes*		1.7 ± 0.4		
*Monocytes*		0.5 ± 0.1		
*Eosinophils*		0.2 ± 0.1		
*Basophils*		0.04 ± 0.02		
*Neutrophils %*		55.7 ± 6		
*Lymphocytes %*		31.3 ± 4.8		
*Monocytes %*		8.6 ± 2.0		
*Eosinophils %*		3.6 ± 2.0		
*Basophils %*		0.8 ± 0.3		
IgE, KUA/l, mean ± SD		267.2 ± 244.3		
IgE, median (Q1, Q3)		173 (120–414)		

Continuous variables are expressed as mean ± standard deviation (SD). *p*-value from Pearson’s chi-square test for continuous variables and *p*-value from the Mann–Whitney U-test or Fisher’s exact test for categorical variables. One subject did not answer the question about children.

**Table 2:** NTA and CF analyses of EVs median concentrations of AR and HS groups.

EVs Subtype	HS Median (Q1; Q3)	AR Median (Q1; Q3)	*p*-Value
***NTA Analysis***			
*Total EVs × 10^6^/mL*	968.1 (803.5; 1267.3)	1267.3 (1031.1; 1585.6)	**0.048**
***Flow cytometry***			
*LTA+*	7.7 (5.8; 9.3)	14 (6.3; 17.7)	0.144
*LPS+*	90 (43.5; 144.8)	167.8 (66.4; 327.5)	0.153
*EpCAM + EVs*	10 (6.6; 12.6)	11.7 (7.1; 13.8)	0.668
*CD294 + EVs*	12 (8.8; 24.6)	17.5 (13.7; 31.6)	**0.019**
*CD203C + EVs*	7 (5.6; 10.3)	12.4 (10.2; 21.1)	**<0.001**
*CD177 + EVs*	16.5 (11.3; 22.1)	18.4 (12.9; 24.2)	0.416
*CD62E + EVs*	19.6 (10.8; 33.9)	19.9 (14.5; 38.7)	0.272
*CD61 + EVs*	130.7 (73.5; 222.2)	178.3 (118.2; 357.2)	0.059
*CD14 + EVs*	23.3 (11.5; 39.4)	28.4 (17.7; 48.7)	0.109

Median with first (Q1) and third (Q3) quartiles and the Mann–Whitney U test for the difference between HS and AR. Bold *p*-values denote a significant difference between the cases and controls (*p*-value < 0.05).

**Table 3:** Association between PM_10_ and PM_2.5_ exposure (day -0) and EVs released by different cell types.

Exposure	EVs Cellular Origin	HS		AR	
		β (95% CI)	*p*-Value	β (95% CI)	*p*-Value
PM_10 (day -0)_	**NTA analysis**				
	*Total EVs*	−0.10 (−0.25; 0.05)	0.173	−0.08 (−0.24; 0.09)	0.353
	**Flow cytometry**				
	*LTA+*	−0.38 (−0.68; −0.08)	**0.013**	0.03 (−0.25; 0.32)	0.829
	*LPS+*	−0.38 (−0.71; −0.05)	**0.023**	0.06 (−0.25; 0.36)	0.721
	*EpCAM + EVs*	0.09 (−0.06; 0.24)	0.241	−0.08 (−0.26; 0.09)	0.358
	*CD294 + EVs*	−0.03 (−0.20; 0.15)	0.752	−0.07 (−0.25; 0.11)	0.450
	*CD203C + EVs*	−0.16 (−0.36; 0.04)	0.109	0.01 (−0.19; 0.18)	0.983
	*CD177 + EVs*	0.08 (−0.12; 0.28)	0.415	−0.03 (−0.24; 0.19)	0.815
	*CD62E + EVs*	0.05 (−0.16; 0.26)	0.671	0.08 (−0.14; 0.30)	0.453
	*CD61 + EVs*	−0.12 (−0.51; 0.27)	0.550	−0.23 (−0.56; 0.11)	0.182
	*CD14 + EVs*	−0.44 (−0.80; −0.09)	**0.013**	−0.15 (−0.46; 0.16)	0.341
PM_2.5 (day -0)_	**NTA analysis**				
	*Total EVs*	−0.08 (−0.8; 0.64)	0.834	0.09 (−0.32; 0.5)	0.668
	**Flow cytometry**				
	*LTA+*	−1.05 (−2.64; 0.54)	0.197	0.30 (−0.40; 1.0)	0.405
	*LPS+*	−0.44 (−2.06; 1.19)	0.598	0.09 (−0.67; 0.85)	0.825
	*EpCAM + EVs*	0.07 (−0.73; 0.86)	0.872	0.01 (−0.41; 0.42)	0.986
	*CD294 + EVs*	0.47 (−0.38; 1.32)	0.281	−0.11 (−0.51; 0.30)	0.608
	*CD203C + EVs*	0.51 (−0.40; 1.42)	0.275	0.22 (−0.18; 0.63)	0.277
	*CD177 + EVs*	0.56 (−0.39; 1.51)	0.244	−0.25 (−0.76; 0.26)	0.330
	*CD62E + EVs*	0.16 (−0.91; 1.23)	0.768	−0.29 (−0.84; 0.25)	0.292
	*CD61 + EVs*	1.58 (−0.28; 3.45)	0.096	0.13 (−0.60; 0.86)	0.730
	*CD14 + EVs*	−1.35 (−3.03; 0.32)	0.113	−0.08 (−0.79; 0.64)	0.835

Adjusted for age, gender, rhinitis status, and interactions between allergic rhinitis condition and PM exposure. Beta represents the change in the EV count for a 10 μg/m^3^ increase in PM concentration. Bold *p*-values denote a significant difference (*p*-value < 0.05).

**Table 4:** α-diversity index in the HS and AR groups.

α-Diversity Index	HS	AR	*p*-Value
	Mean ± SD	Mean ± SD	
*Observed species*	99.6 ± 19.5	81.4 ± 25.4	**0.009**
*Chao 1*	118.8 ± 34.0	98.2 ± 24.7	**0.023**
*PD whole tree*	7.5 ± 1.2	6.4 ± 1.5	**0.009**
*Shannon*	3.2 ± 0.93	2.98 ± 0.98	0.370
*Simpson*	0.75 ± 0.17	0.73 ± 0.20	0.674

Mean with standard deviation (SD) and two-tailed *t*-test for the difference between HS and AR. Bold *p*-values denote a significant difference (*p*-value < 0.05).

**Table 5:** Factor-loading matrix, communalities, and explained variance for two microbiome patterns identified by factor analysis.

Genera	Factor1	Factor2	Communalities
*Abiotrophia*	0.45	-	0.20
*Acinetobacter*	0.32	**0.71**	0.60
*Actinomyces*	**0.69**	-	0.48
*Anaerococcus*	−0.18	**0.75**	0.59
*Capnocytophaga*	0.57	-	0.33
*Citrobacter*	-	0.41	0.17
*Dialister*	0.14	0.51	0.28
*Fusobacterium*	**0.65**	0.11	0.43
*Granulicatella*	0.59	-	0.35
*Leptotrichia*	0.58	0.18	0.37
*Peptoniphilus*	-	**0.71**	0.50
*Porphyromonas*	0.54	0.14	0.31
*Prevotella*	0.39	0.29	0.23
*Propionibacterium*	0.25	0.60	0.42
*Pseudomonas*	-	0.57	0.33
*Rothia*	**0.68**	-	0.46
*Staphylococcus*	0.23	**0.70**	0.55
*Streptococcus*	**0.72**	0.36	0.64
*Veillonella*	0.52	0.28	0.35
Proportion of explained variance (%)	0.44	0.22	
Cumulative explained variance (%)	0.44	0.66	

Estimated from a factor analysis performed on 19 genera. The magnitude of each loading indicates the importance of the corresponding genera to the factor. Loading ≥ 0.63 were shown in bold typeface; loading < |0.10| were suppressed.

**Table 6:** Association between PM exposure (day -5) and α-diversity indices.

PM Exposure	α-Diversity Index	HS		AR	
		β (95% CI)	*p*-Value	β (95% CI)	*p*-Value
PM_10 (day -5)_	*Observed species*	−7.6 (−17.3; 2.1)	0.122	8.6 (−0.7; 18)	0.070
	*Chao1*	−7.2 (−21.3; 6.9)	0.307	7.8 (−5.8; 21.3)	0.254
	*PD whole tree*	−0.3 (−1.0; 0.3)	0.284	0.4 (−0.2; 1.0)	0.197
	*Shannon*	−0.2 (−0.7; 0.2)	0.325	0.3 (−0.1; 0.8)	0.114
	*Simpson*	0.0 (−0.1; 0.1)	0.465	0.1 (0.0; 0.1)	0.102
PM_2.5(day -5)_	*Observed species*	−22.5 (−43; −2)	**0.032**	34.2 (8.9; 59.6)	**0.010**
	*Chao1*	−25.3 (−55.4; 4.8)	0.097	36.7 (−0.6; 74.0)	0.053
	*PD whole tree*	−1.1 (−2.4; 0.2)	0.104	1.8 (0.1; 3.4)	**0.036**
	*Shannon*	−0.97 (−1.89; 0.04)	**0.041**	1.2 (0.1; 2.4)	**0.040**
	*Simpson*	−0.2 (−0.4; −0.1)	**0.012**	0.2 (0.0; 0.4)	0.089

Adjusted for age, gender, smoking habit, mean temperature, mean humidity, case-control condition, and the interactions between case-control and PM exposure. β represents the change in the α-diversity index for a 10 μg/m^3^ increase in PM concentration. Bold *p*-values denote a significant difference (*p*-value < 0.05).

**Table 7:** Association between PM and EVs subtypes for the selected values of Factor1, which is characterized by the greatest positive loadings on Streptococcus, Actinomyces, Rothia, and Fusobacterium.

Outcome: EVs Subtypes	Level of Factor1	β _PM10_ (95% CI)	*p*-Value	*	β _PM2.5_ (95% CI)	*p*-Value	*p*-Value *
***NTA analysis***							
***Total EVs × 10^6^/mL***	p5	−0.02 (−0.20, 0.15)	0.792	0.351	0.01 (−0.51, 0.54)	0.966	0.899
	Median	−0.10 (−0.22, 0.02)	0.107		0.05 (−0.3, 0.41)	0.762	
	p95	−0.18 (−0.4, 0.04)	0.110		0.10 (−0.57, 0.77)	0.770	
***Flow cytometry***							
***LTA+***	p5	0.21 (−0.10, 0.52)	0.191	**0.008**	0.51 (−0.36, 1.39)	0.251	0.277
	Median	−0.22 (−0.45, 0.02)	0.068		0.03 (−0.64, 0.7)	0.928	
	p95	−0.66 (−1.11, −0.2)	**0.004**		−0.47 (−1.76, 0.83)	0.480	
***LPS+***	p5	0.27 (−0.02, 0.55)	0.067	**0.001**	0.89 (−0.12, 1.91)	0.085	**0.034**
	Median	−0.21 (−0.43, 0.02)	0.072		−0.09 (−0.79, 0.61)	0.807	
	p95	−0.69 (−1.1, −0.28)	**0.001**		−1.10 (−2.34, 0.15)	0.085	
***EpCAM + EVs***	p5	0.14 (−0.03, 0.31)	0.117	0.073	−0.09 (−0.61, 0.44)	0.745	0.561
	Median	0.01 (−0.13, 0.12)	0.995		0.05 (−0.31, 0.42)	0.778	
	p95	−0.14 (−0.36, 0.08)	0.207		0.2 (−0.47, 0.86)	0.562	
***CD294 + EVs***	p5	0.03 (−0.16, 0.23)	0.727	0.283	−0.12 (−0.64, 0.39)	0.640	0.460
	Median	−0.06 (−0.20, 0.08)	0.391		0.06 (−0.33, 0.44)	0.766	
	p95	−0.16 (−0.41, 0.09)	0.217		0.24 (−0.47, 0.96)	0.502	
***CD203C + EVs***	p5	0.02 (−0.18, 0.22)	0.833	0.180	0.47 (−0.05, 0.99)	0.075	0.345
	Median	−0.11 (−0.26, 0.05)	0.180		0.24 (−0.15, 0.63)	0.236	
	p95	−0.24 (−0.53, 0.05)	0.102		0.01 (−0.72, 0.72)	0.991	
***CD177 + EVs***	p5	0.12 (−0.11, 0.35)	0.310	0.738	−0.14 (−0.81, 0.54)	0.689	0.901
	Median	0.03 (−0.13, 0.20)	0.682		−0.03 (−0.51, 0.45)	0.899	
	p95	−0.06 (−0.35, 0.24)	0.711		0.08 (−0.82, 0.98)	0.863	
***CD62E + EVs***	p5	0.29 (0.07, 0.50)	**0.008**	**0.004**	−0.07 (−0.79, 0.65)	0.852	0.613
	Median	0.01 (−0.16, 0.16)	0.994		−0.23 (−0.74, 0.29)	0.383	
	p95	−0.3 (−0.58, −0.01)	**0.040**		−0.39 (−1.29, 0.50)	0.390	
***CD61 + EVs***	p5	−0.14 (−0.54, 0.26)	0.494	0.510	0.51 (−0.52, 1.53)	0.332	0.287
	Median	−0.22 (−0.51, 0.07)	0.140		0.09 (−0.86, 1.04)	0.853	
	p95	−0.30 (−0.84, 0.23)	0.264		−0.34 (−2.3, 1.62)	0.734	
***CD14 + EVs***	p5	0.21 (−0.09, 0.51)	0.163	**0.001**	0.69 (−0.16, 1.54)	0.112	**0.013**
	Median	−0.32 (−0.55, −0.09)	**0.006**		−0.36 (−0.99, 0.27)	0.264	
	p95	−0.87 (−1.32, −0.43)	**0.000**		−1.44 (−2.6, −0.27)	**0.016**	

p5, 5th percentile; p95, 95th percentile. β are calculated from negative binomial regression models adjusted for age, gender, smoking habit, mean temperature, mean humidity, allergy status (HS or AR), PM _(day -0)_, and the interactions between microbiome Factor1 and PM exposure, and represents the change in the α-diversity index for a 10 μg/m^3^ increase in PM concentration. * *p*-value for interaction. Bold *p*-values denote a significant difference (*p*-value < 0.05).

**Table 8:** Association between PM and EVs subtypes for the selected values of Factor2, which is characterized by the greatest positive loadings on Anaerococcus, Acinetobacter, Peptoniphilus, and Staphylococcus.

Outcome: EVs Subtypes	Level of Factor2	β _PM10_ (95% CI)	*p*-Value	*	β _PM2.5_ (95% CI)	*p*-Value	*p*-Value *
***NTA analysis***							
***Total EVs total × 10^6^/mL***	p5	−0.09 (−0.32, 0.15)	0.481	0.842	0.01 (−0.52, 0.55)	0.957	0.846
	Median	−0.10 (−0.22, 0.01)	0.085		−0.04 (−0.43, 0.36)	0.853	
	p95	−0.11 (−0.29, 0.06)	0.206		−0.07 (−0.64, 0.51)	0.824	
***Flow cytometry***							
***LTA+***	p5	−0.22 (−0.69, 0.24)	0.341	0.657	0.59 (−0.42, 1.6)	0.253	0.369
	Median	−0.12 (−0.36, 0.11)	0.301		0.05 (−0.70, 0.8)	0.896	
	p95	−0.07 (−0.44, 0.3)	0.708		−0.24 (−1.45, 0.97)	0.698	
***LPS+***	p5	−0.56 (−0.99, −0.12)	**0.012**	**0.029**	−0.43 (−1.49, 0.63)	0.429	0.269
	Median	−0.09 (−0.33, 0.14)	0.441		0.34 (−0.43, 1.11)	0.390	
	p95	0.16 (−0.19, 0.51)	0.380		0.75 (−0.57, 2.07)	0.265	
***EpCAM + EVs***	p5	0.08 (−0.19, 0.35)	0.559	0.633	−0.01 (−0.62, 0.61)	0.985	0.861
	Median	0.02 (−0.11, 0.15)	0.759		0.05 (−0.36, 0.47)	0.807	
	p95	−0.01 (−0.21, 0.18)	0.894		0.08 (−0.56, 0.73)	0.800	
***CD294 + EVs***	p5	0.02 (−0.25, 0.28)	0.907	0.718	0.10 (−0.49, 0.69)	0.736	0.778
	Median	−0.03 (−0.15, 0.1)	0.651		0.20 (−0.19, 0.58)	0.323	
	p95	−0.05 (−0.24, 0.14)	0.579		0.25 (−0.38, 0.87)	0.440	
***CD203C + EVs***	p5	−0.06 (−0.36, 0.24)	0.693	0.943	0.59 (−0.05, 1.23)	0.072	0.389
	Median	−0.07 (−0.22, 0.08)	0.359		0.28 (−0.15, 0.7)	0.205	
	p95	−0.08 (−0.3, 0.15)	0.505		0.10 (−0.59, 0.8)	0.767	
***CD177 + EVs***	p5	0.01 (−0.35, 0.37)	0.956	0.831	−0.70 (−1.46, 0.07)	0.076	**0.042**
	Median	0.05 (−0.12, 0.21)	0.582		0.19 (−0.31, 0.69)	0.455	
	p95	0.06 (−0.18, 0.31)	0.602		0.67 (−0.13, 1.46)	0.099	
***CD62E + EVs***	p5	0.2 (−0.14, 0.55)	0.254	0.472	−0.34 (−1.18, 0.51)	0.437	0.459
	Median	0.08 (−0.08, 0.24)	0.307		0.02 (−0.53, 0.57)	0.935	
	p95	0.02 (−0.22, 0.26)	0.874		0.22 (−0.69, 1.12)	0.639	
***CD61 + EVs***	p5	−0.17 (−0.66, 0.31)	0.484	0.977	0.15 (−1.07, 1.36)	0.815	0.706
	Median	−0.18 (−0.47, 0.11)	0.228		0.45 (−0.49, 1.4)	0.350	
	p95	−0.18 (−0.64, 0.27)	0.430		0.62 (−1.01, 2.24)	0.458	
***CD14 + EVs***	p5	−0.25 (−0.73, 0.24)	0.316	0.871	0.55 (−0.58, 1.67)	0.342	0.207
	Median	−0.21 (−0.46, 0.04)	0.102		−0.33 (−1.12, 0.47)	0.423	
	p95	−0.19 (−0.57, 0.19)	0.329		−0.8 (−2.17, 0.57)	0.254	

p5, 5th percentile; p95, 95th percentile. β are calculated from negative binomial regression models adjusted for age, gender, smoking habit, mean temperature, mean humidity, allergy status (HS or AR), PM _(day -0)_, and the interactions between microbiome Factor2 and PM exposure, and represents the change in the α-diversity index for a 10 μg/m^3^ increase in PM concentration. *, *p*-value for interaction. Bold *p*-values denote a significant difference (*p*-value < 0.05).

## Data Availability

All data needed to evaluate the conclusions in the paper are present in the paper and/or the Supplementary Materials. Raw reads data have been submitted to the GeneBank databases under BioProject accession number PRJNA646474. Additional data related to this paper may be requested to the authors.

## Supplementary Materials

The following are available online at https://www.mdpi.com/article/10.3390/ijerph182010689/s1, Table S1: MISEV 2018 guidelines compliance; Table S2: Genera description for the factor analysis; Table S3: Factorability of the correlation matrix of the original variables; Figure S1: LTA + (gram-positive) EVs gating protocols for plasmatic EVs; LPS + (gram-negative) EVs gating protocols for plasmatic EVs; and CD294 + EVs gating protocols for plasmatic EVs; Figure S2: LPS + (GRAM-negative) EVs gating protocols for plasmatic EVs; Figure S3: CD294+ EVs gating protocols for plasmatic EVs; Figure S4: CD203 + EVs gating protocols for plasmatic EVs; Figure S5: PM_10_ and PM_2.5_ daily exposure levels; and Figure S6: bNM diversity.
