# Supplementary material for: Particulate Matter Exposure and Allergic Rhinitis: The Role of Plasmatic Extracellular Vesicles and Bacterial Nasal Microbiome
Source: Int J Environ Res Public Health. 2021 Oct 12;18(20):10689. doi: 10.3390/ijerph182010689 (PMC8535327; doi:10.3390/ijerph182010689)
Supplement: Supplementary file 1 [file ijerph-18-10689-s001.zip › ijerph-1366401-supplementary.pdf]

Supplementary Table S1

MISEV 2018 guidelines compliance

	**Section title**	**Required information according to MISEV2018**	**Mandatory requirement**	**Not applicable/not available**	**Our approach**	**Compliance with MISEV2018 requirements**
1	Nomenclature	The term extracellular vesicle (EV) can be used with demonstration of extracellular (no intact cells) and vesicular nature per these characterizations and function	YES		As explained in sections 4 and 5, the term extracellular vesicle (EV) has been used in the manuscript	YES
2a	Collection and pre-processing (tissue culture conditioned medium)	General cell characterization	YES	N/A		
2a	Collection and pre-processing (tissue culture conditioned medium)	Medium used before and during collection (additives, serum, other)	YES	N/A		
2a	Collection and pre-processing (tissue culture conditioned medium)	Exact protocol for the depletion of EVs from additives in collection medium	YES	N/A		

2a	Collection and pre-processing (tissue culture conditioned medium)	Nature and size of culture vessels, and volume of the medium during conditioning<br>e) specific culture conditions (treatment, % O2, coating,polarization…) before and during collection	N/A			
2a	Collection and pre-processing (tissue culture conditioned medium)	Number of cells/ml and % of live/ dead cells at time of collection	N/A			
2a	Collection and pre-processing (tissue culture conditioned medium)	Frequency and interval of Conditioned Medium harvest	N/A			
2b and 2c	Collection and pre-processing (Biofluids or tissues)	Donor status if available (age, sex, food/water<br>intake, collection time, disease, medication, other)	YES		Each recruited subject filled in an informative questionnaire before blood drawing; blood samples were processed within 2 hrs after blood draws	YES
2b and 2c	Collection and pre-processing (Biofluids or tissues)	Volume of biofluid or volume/mass of tissue sample<br>collected per donor	YES		For each subject, two blood samples were collected in two EDTA tubes (7 mL each)	YES

2b and 2c	Collection and pre-processing (Biofluids or tissues)	Total volume/mass used for EV isolation (if pooled from several donors)	YES		EVs isolation was performed starting from two aliquots of 3 mL of plasma for each subject. Each EV pellet aliquot obtained from each subject was resuspended in 1 mL of PBS triple filtered (pore size 0.1 µm).	YES
2b and 2c	Collection and pre-processing (Biofluids or tissues)	All known collection conditions, including additives, at time of collection			Blood samples were collected in 7 mL EDTA tubes	YES
2b and 2c	Collection and pre-processing (Biofluids or tissues)	Pre-treatment to separate major fluid-specific contaminants before EV isolation			Blood was centrifuged at 1200 × *g* for 15 min at room temperature to obtain platelet-free plasma. After that, two aliquots of 3 mL of plasma for each subject were subsequently centrifuged at 1000, 2000, and 3000 × *g* for 15 min at 4 °C. The obtained pellets were discarded to remove cell debris.	YES
2b and 2c	Collection and pre-processing (Biofluids or tissues)	Temperature and time of biofluid/tissue handling before and during pre-treatment			Each sample was processed within 2 hrs and maintained at 4 °C	YES
2b and 2c	Collection and pre-processing (Biofluids or tissues)	For cultured tissue explants: volume, nature of		N/A		

		medium and time of culture before collecting conditioned<br><br>medium				
2b and 2c	Collection and pre-processing (Biofluids or tissues)	For direct tissue EV extraction: treatment of tissue<br><br>to release vesicles without disrupting cells		N/A		
2d	Storage and recovery	Storage and recovery (e.g., thawing) of CCM, biofluid, or tissue before EV isolation (storage temperature, vessel, time; method of thawing or other sample preparation)	N/A			
2d	Storage and recovery	Storage and recovery of EVs after isolation (temperature, vessel, time, additive(s)…)	YES		Isolated EVs were immediately characterized by flow cytometry and Nanosight.	YES
3	EV separation and concentration	Centrifugation: reference number of tube(s), rotor(s), adjusted k factor(s) of each centrifugation step (= time+<br><br>speed+ rotor, volume/density of centrifugation conditions), temperature, brake settings	YES		We applied differential ultracentrifugation preceded by lower-speed centrifugation steps. In particular, 7 mL of whole blood were collected in Corning 15 mL PP Centrifuge Tubes and centrifuged at 1000, 2000, and 3000 × g for 15 min at 4 °C (Haereus Labofuge 400R, Hanau, Germany). The obtained pellets were discarded to remove	YES

					cells, apoptotic bodies, and cell debris. EVs were then isolated from supernatants by ultracentrifugation at 110,000 × g for 94 min at 4 °C in polypropylene ultracentrifuge tubes (Quick-Seal ultra-clear centrifuge tubes, Beckman Coulter; Brea, CA, USA) rotor MLA-55 (Beckman Coulter), filled with PBS previously filtered through a 0.10-µm pore-size polyethersulfone filter (StericupRVP, Merck Millipore; Burlington, MA, USA). Our method is included in the category "Intermediate recovery, intermediate specificity = mixed EVs with limited non-EV components"	
3	EV separation and concentration	Density gradient: nature of matrix, method of generating gradient, reference (and size) of tubes, centrifugation speed and time (with brake specified), method, and volume of fraction recovery.		N/A		
3	EV separation and concentration	Chromatography: matrix (nature, pore size,…), loaded sample volume, fraction volume, number		N/A		

3	EV separation and concentration	Precipitation: reference of polymer, ratio vol/vol or weight/vol polymer/fluid, time/temperature of incubation, time/speed/temperature of centrifugation		N/A		
3	EV separation and concentration	Filtration: reference of filter type (=nature of the membrane, pore size…), time and speed of centrifugation, volume before/after (in case of concentration)		N/A		
3	EV separation and concentration	Antibody-based characterization: reference of antibodies, mass Ab/amount of EVs, nature of Ab carrier (bead, surface), and amount of Ab/carrier surface		N/A		
3	EV separation and concentration	Other: all necessary details to allow replication		N/A		
3	EV separation and concentration	Additional step(s) to concentrate, if any		N/A		
3	EV separation and concentration	Additional step(s) to wash matrix and/or sample, if any		N/A		

4a	EV characterization, Quantification	Volume of fluid, and/or cell number, and/or tissue mass used to isolate EVs	N/A			
4a	EV characterization, Quantification	Global quantification by at least 2 methods: protein amount, particle number, lipid amount, expressed per volume of initial fluid or number of producing cells/mass of tissue	YES		To quantify the total number of EVs we applied the two following approaches:<br><br>1) Nanoparticle tracking analysis by NanoSight NS300 system (Malvern Panalytical Ltd, Malvern, UK). Five 30-s recordings were made for each sample. Collected data were analyzed with NTA software (Malvern Panalytical Ltd.), which provided high-resolution particle-size distribution profiles as well as measurements of the EV concentration.<br><br>2) High-resolution Flow cytometry by MACSQuant, Miltenyi Biotec. To analyze EV integrity, 60 µl aliquots were stained with 0.2 µM 5(6)-carboxyfluorescein diacetate N-succinimidyl ester (CFSE) at 37 °C for 20 min in the dark. CFSE is a cell-permeant, non-fluorescent pro-dye. If incorporated into intact EVs, which contain esterases as live cells, the acetate groups of CFSE are cleaved producing a membrane-impermeant molecule with green fluorescence.	YES

4a	EV characterization, Quantification	Ratio of the 2 quantification figures	YES	N/A		
4b	EV characterization, General Characterization	At least <u>three</u> positive protein markers of EVs, including at least one transmembrane/lipid-bound protein and one cytosolic protein<br><br>At least one negative protein marker	YES		The following antibodies have been used to measure positive protein markers of plasmatic EVs:<br><br>AbCD177-APC (neutrophils, clone REA 598; Miltenyi Biotec), AbCD14-APC (monocytes, clone TUK4, Miltenyi Biotec), AbCD61-APC (platelets, clone Y2/51, Miltenyi Biotec), CD326 (EpCAM)-APC (epithelium, clone HEA-125, Miltenyi Biotec), AbCD62E-APC (activated epithelium, clone REA280, Miltenyi Biotec), AbCD203C (mast cells, clone REA 826, Miltenyi Biotec), AbCD294 (eosinophils, clone REA 598, Miltenyi Biotec); Ab-Lipopolysaccharide (LPS) (Invitrogen, Waltham, MA, USA), unconjugated anti-Lipoteichoic acid (Invitrogen), secondary IgG-antimouse (IgG (H+L), Superclonal™ Recombinant Secondary Antibody, Alexa Fluor 647- Invitrogen).<br><br><u>Transmembrane, tissue-specific, and focus of the present study.</u><br><br>CFSE staining was used to assess cytosolic esterase activity. As a negative control, representative	YES

					EVs samples were incubated with Tween20 (P1379; Merk, Sigma-Aldrich) ON at 37 °C, and then incubated with CFSE as described above. Positivity for CFSE fluorescence was evaluated by High-Resolution Flow cytometry analysis	
4c	Single EV characterization	Images of single EVs by electron microscopy	YES		Transmission Electron Microscopy (TEM) analysis was performed on random samples as quality control.	YES
4c	Single EV characterization	Non-image-based method analyzing large numbers of single EVs: Non-image-based method analyzing large numbers of single EVs: NTA, TRPS, FCS, high-resolution flow cytometry, multi-angle light-scattering, Raman spectroscopy, etc.	YES		NTA and Flow Cytometry were performed	YES
5	Functional studies	Dose-response assessment	N/A			
5	Functional studies	Negative control = nonconditioned medium, biofluid/tissue from control donors, as applicable	N/A			
5	Functional studies	Quantitative comparison of functional activity of total fluid, vs EV-depleted fluid,	N/A			

		vs EVs (after high recovery/low specificity separation)				
5	Functional studies	Quantitative comparison of functional activity of EVs vs other EPs/fractions after low recovery/high specificity separation	N/A			
5	Functional studies	Quantitative comparison of the activity of EV subtypes (if subtype-specific function claimed)	N/A			
5	Functional studies	The extent of functional activity in the absence of contact between EV donor and EV recipient	N/A			
6	Reporting	Submission of data (proteomics, sequencing, other) to relevant public, curated databases or open-access repository	YES	N/A		

Supplementary Table S2. Genera description for Factor Analysis. *Description of the 81 genera, frequencies of non-zero relative abundance, and reason for the exclusion of 61 genera from the factor analysis.*

Genera	**Mean ± SD**	**Median [Q1; Q3]**	**Number of subjects with non-zero relative abundance (%)**	**Reason for exclusion from FA**
Prevotella	0.19 ± 0.78	0.01 [0; 0.05]	29 (65)	"unassigned", and "other"
Abiotrophia	0.09 ± 0.3	0 [0; 0.03]	20 (46)	
Acinetobacter	0.45 ± 0.81	0.15 [0.04; 0.48]	43 (96)	
Actinobacillus	0.04 ± 0.09	0 [0; 0.03]	18 (41)	Mean ≤ 0.04 and median ≤ 0.03
Actinomyces	0.25 ± 0.77	0.03 [0.01; 0.12]	38 (85)	
Aerococcus	0.02 ± 0.06	0 [0; 0]	13 (30)	Mean ≤ 0.04 and median ≤ 0.03
Aggregatibacter	0.1 ± 0.31	0 [0; 0.02]	25 (57)	Communalities < 0.15
Alloiococcus	6.18 ± 12.38	0.39 [0.04; 6.67]	45 (100)	KMO < 0.30
Anaerococcus	1.79 ± 1.93	1.29 [0.52; 2.78]	45 (100)	
Bifidobacterium	0.03 ± 0.2	0 [0; 0]	0 (2)	Mean ≤ 0.04 and median ≤ 0.03
Brachybacterium	0.02 ± 0.04	0 [0; 0.01]	18 (41)	Mean ≤ 0.04 and median ≤ 0.03
Brevibacterium	0.05 ± 0.14	0 [0; 0.02]	18 (41)	KMO < 0.30
Brochothrix	0.17 ± 1	0 [0; 0.01]	13 (30)	KMO < 0.30
Bulleidia	0.05 ± 0.21	0 [0; 0]	6 (15)	No correlation
Campylobacter	0.06 ± 0.14	0 [0; 0.03]	23 (52)	KMO < 0.30
Capnocytophaga	0.06 ± 0.15	0 [0; 0.02]	22 (50)	
Cardiobacterium	0.01 ± 0.05	0 [0; 0.01]	12 (28)	Mean ≤ 0.04 and median ≤ 0.03
Carnobacterium	0.03 ± 0.08	0 [0; 0.02]	22 (50)	Mean ≤ 0.04 and median ≤ 0.03
Chryseobacterium	0.05 ± 0.14	0 [0; 0.02]	19 (43)	KMO < 0.30
Citrobacter	0.06 ± 0.17	0 [0; 0.02]	20 (46)	
Cloacibacterium	0.03 ± 0.07	0 [0; 0.02]	22 (50)	Mean ≤ 0.04 and median ≤ 0.03
Corynebacterium	36.1 ± 24.1	29.9 [18.6; 51.6]	45 (100)	KMO < 0.30
Deinococcus	0.02 ± 0.08	0 [0; 0]	2 (7)	Mean ≤ 0.04 and median ≤ 0.03
Delftia	0.03 ± 0.04	0 [0; 0.03]	24 (54)	Mean ≤ 0.04 and median ≤ 0.03
Dialister	0.15 ± 0.51	0 [0; 0.03]	23 (52)	
Enhydrobacter	0.27 ± 0.48	0.13 [0.02; 0.3]	41 (91)	KMO < 0.30
Escherichia	0.08 ± 0.16	0.01 [0; 0.06]	33 (74)	KMO < 0.30
Finegoldia	0.58 ± 0.98	0.14 [0.01; 0.66]	40 (89)	KMO < 0.30
Fusobacterium	0.16 ± 0.3	0.04 [0; 0.11]	37 (83)	
Gordonia	0.07 ± 0.37	0 [0; 0.01]	16 (37)	KMO < 0.30
Granulicatella	0.18 ± 0.54	0.02 [0; 0.15]	37 (83)	
Haemophilus	0.19 ± 0.28	0.07 [0.01; 0.24]	42 (93)	KMO < 0.30
Janthinobacterium	0.14 ± 0.66	0.01 [0; 0.05]	33 (74)	Communalities < 0.15
Kingella	0.02 ± 0.06	0 [0; 0]	15 (35)	Mean ≤ 0.04 and median ≤ 0.03
Kocuria	0.24 ± 0.39	0.1 [0.01; 0.26]	39 (87)	KMO < 0.30
Lactobacillus	0.06 ± 0.28	0 [0; 0]	16 (37)	Communalities < 0.15
Lautropia	0.03 ± 0.08	0 [0; 0.03]	23 (52)	Mean ≤ 0.04 and median ≤ 0.03
Leptotrichia	0.09 ± 0.37	0 [0; 0.02]	22 (50)	
Methylobacterium	0.02 ± 0.06	0 [0; 0.01]	16 (37)	Mean ≤ 0.04 and median ≤ 0.03
Microbacterium	0.03 ± 0.06	0.01 [0; 0.04]	32 (72)	Mean ≤ 0.04 and median ≤ 0.03
Microbispora	0.37 ± 2.44	0 [0; 0]	16 (37)	KMO < 0.30
Micrococcus	0.21 ± 0.3	0.09 [0.02; 0.26]	42 (93)	Communalities < 0.15
Modestobacter	0.03 ± 0.12	0 [0; 0.01]	14 (33)	Mean ≤ 0.04 and median ≤ 0.03
Moraxella	9.86 ± 24.06	0.08 [0.02; 0.28]	44 (98)	KMO < 0.30
Neisseria	0.52 ± 1.69	0.04 [0.01; 0.27]	43 (96)	KMO < 0.30
Other_Enterobacteriaceae	0.37 ± 0.87	0.04 [0.01; 0.17]	39 (87)	"unassigned", and "other"

Other_Microbacteriaceae	0.01 ± 0.02	0 [0; 0.01]	23 (52)	“unassigned”, and “other”
Other_Pseudomonadaceae	0.02 ± 0.06	0 [0; 0.02]	23 (52)	“unassigned”, and “other”
Paludibacter	0.01 ± 0.07	0 [0; 0]	4 (11)	Mean ≤ 0.04 and median ≤ 0.03
Paracoccus	0.37 ± 0.7	0.11 [0.02; 0.39]	40 (89)	KMO < 0.30
Parvimonas	0.04 ± 0.23	0 [0; 0.01]	13 (30)	Communalities < 0.15
Peptoniphilus	1.45 ± 1.95	0.7 [0.07; 2.59]	45 (100)	
Phycicoccus	0.04 ± 0.08	0 [0; 0.03]	28 (63)	Mean ≤ 0.04 and median ≤ 0.03
Porphyromonas	0.06 ± 0.14	0.01 [0; 0.05]	29 (65)	
Prevotella	0.2 ± 0.47	0.02 [0; 0.2]	33 (74)	
Propionibacterium	7.54 ± 7.94	5.27 [2.36; 9.16]	45 (100)	
Pseudomonas	1.28 ± 2.35	0.29 [0.03; 1.34]	45 (100)	
Psychrobacter	0.02 ± 0.12	0 [0; 0]	3 (9)	Mean ≤ 0.04 and median ≤ 0.03
Psychromonas	0.17 ± 0.97	0 [0; 0]	2 (7)	No correlation
Roseomonas	0.01 ± 0.04	0 [0; 0.01]	13 (30)	Mean ≤ 0.04 and median ≤ 0.03
Rothia	0.52 ± 1.78	0.09 [0.03; 0.45]	40 (89)	
Rubellimicrobium	0.02 ± 0.07	0 [0; 0]	13 (30)	Mean ≤ 0.04 and median ≤ 0.03
Selenomonas	0.04 ± 0.18	0 [0; 0]	12 (28)	Mean ≤ 0.04 and median ≤ 0.03
Serratia	0.02 ± 0.1	0 [0; 0]	3 (9)	No correlation
Sphingomonas	0.06 ± 0.11	0.02 [0; 0.06]	33 (74)	KMO < 0.30
Staphylococcus	18.79 ± 16.93	12.26 [6.41; 30.41]	45 (100)	
Stenotrophomonas	0.02 ± 0.03	0 [0; 0.04]	25 (57)	Mean ≤ 0.04 and median ≤ 0.03
Streptococcus	3.31 ± 6.95	0.7 [0.25; 2.46]	45 (100)	
*Unassigned__Weeksellaceae_*	0.03 ± 0.09	0 [0; 0]	12 (28)	“unassigned”, and “other”
Unassigned_Caulobacteraceae	0.01 ± 0.03	0 [0; 0.01]	18 (41)	“unassigned”, and “other”
Unassigned_Clostridiaceae	0.02 ± 0.04	0 [0; 0]	12 (28)	“unassigned”, and “other”
Unassigned_Clostridiales	0.01 ± 0.03	0 [0; 0]	6 (15)	“unassigned”, and “other”
Unassigned_CW040	0.03 ± 0.2	0 [0; 0]	4 (11)	“unassigned”, and “other”
Unassigned_Gemellaceae	0.27 ± 0.59	0.07 [0.02; 0.19]	40 (89)	“unassigned”, and “other”
Unassigned_Geodermatophilaceae	0.02 ± 0.06	0 [0; 0.01]	18 (41)	“unassigned”, and “other”
Unassigned_Micrococcaceae	0.09 ± 0.23	0.01 [0; 0.05]	37 (83)	“unassigned”, and “other”
Unassigned_Neisseriaceae	5.3 ± 11.74	0.24 [0.03; 3.92]	44 (98)	“unassigned”, and “other”
Unassigned_Streptophyta	0.36 ± 0.44	0.2 [0.08; 0.49]	43 (96)	“unassigned”, and “other”
Unassigned_TM7_3	0.08 ± 0.35	0 [0; 0.01]	14 (33)	“unassigned”, and “other”
Unassigned_Xenococcaceae	0.02 ± 0.06	0 [0; 0]	7 (17)	“unassigned”, and “other”
Veillonella	0.2 ± 0.53	0.05 [0; 0.19]	33 (74)	

SD, standard deviation; Q1, first quartile; Q3 third quartile; KMO: Kaiser-Meyer-Olkin Statistic

Supplementary table S3. ***Factorability of the correlation matrix of the original variables****: individual and overall measures of sampling adequacy and Bartlett's test of sphericity*

**Bartlett's test of sphericity:**	p-value <0.0001
**Kaiser-Meyer-Olkin statistic - Overall measure of sampling adequacy:**	0.62
**Individual meausures of sampling adequacy:**	
0.40 - 0.50	Capnocytophaga
0.50 - 0.60	Prevotella<br>Rothia<br>Leptotrichia<br>Abiotrophia<br>Citrobacter<br>Granulicatella<br>Streptococcus<br>Actinomyces
0.60 - 0.70	Pseudomonas<br>Fusobacterium<br>Peptoniphilus<br>Anaerococcus<br>Porphyromonas
0.70 - 0.80	Dialister<br>Acinetobacter<br>Veillonella<br>Propionibacterium
≥ 0.80	Staphylococcus

Overall and individual measure of sampling adequacy range between 0 and 1, with values >0.50 indicating an acceptable size

Supplementary Figure S1: LTA+ (gram-positive) EVs gating protocols for plasmatic EVs. A-C negative control with PBS; D-F Plasma samples. A, D): scatter plots; B, E): dot plots with gating for CFSE fluorescence; C, F): dot plots for experimental sample (scatter+ CFSE + ANTI-GRAM+ antibody); D: dot plots for scatter+ ANTI-GRAM+ antibody

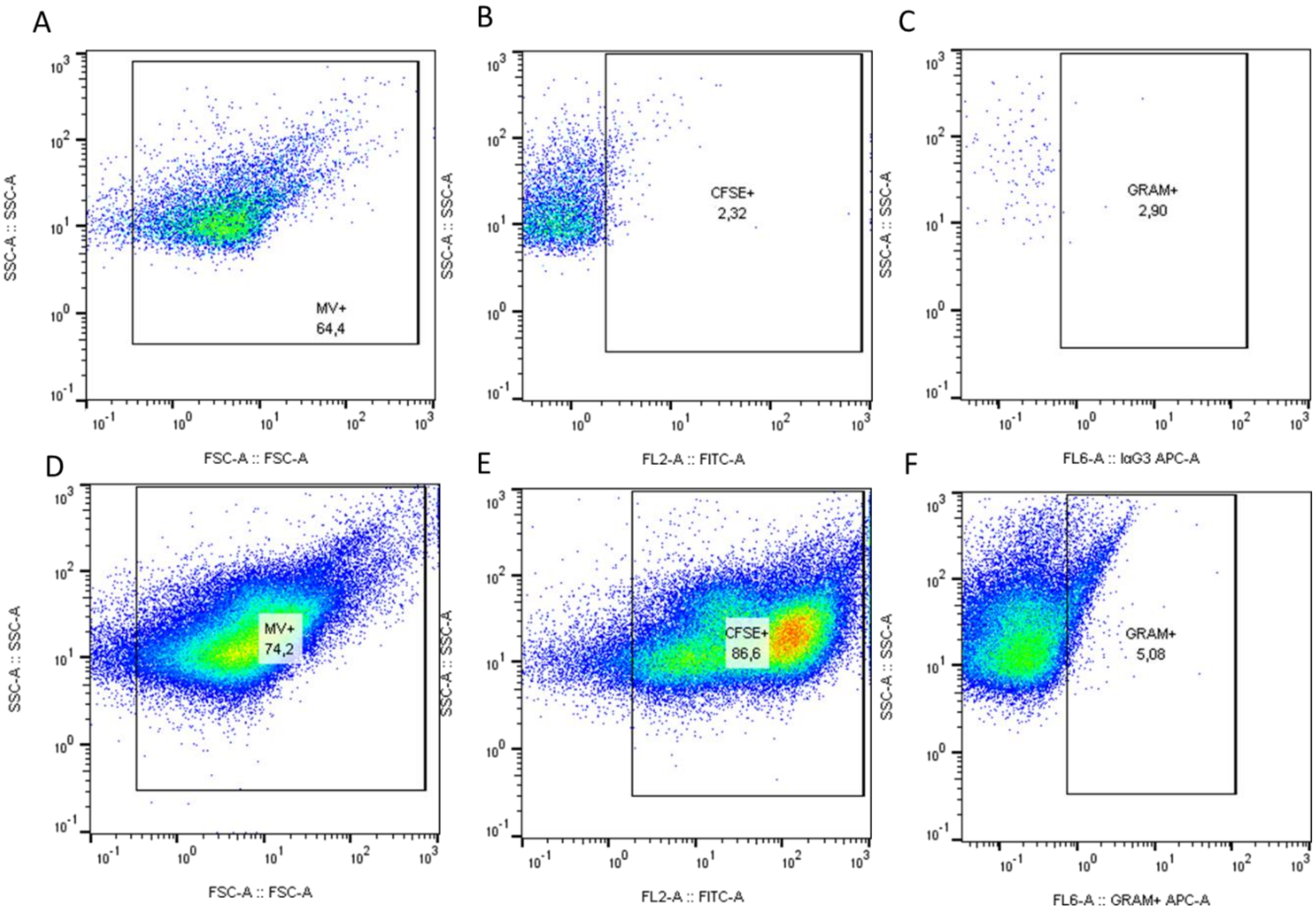

Supplementary Figure S2: LPS+ (GRAM-negative) EVs gating protocols for plasmatic EVs. A-C negative control with PBS; D-F Plasma samples. A, D): scatter plots; B, E): dot plots with gating for CFSE fluorescence; C, F): dot plots for experimental sample (scatter+ CFSE + ANTI-GRAM- antibody); D: dot plots for scatter+ ANTI-GRAM- antibody

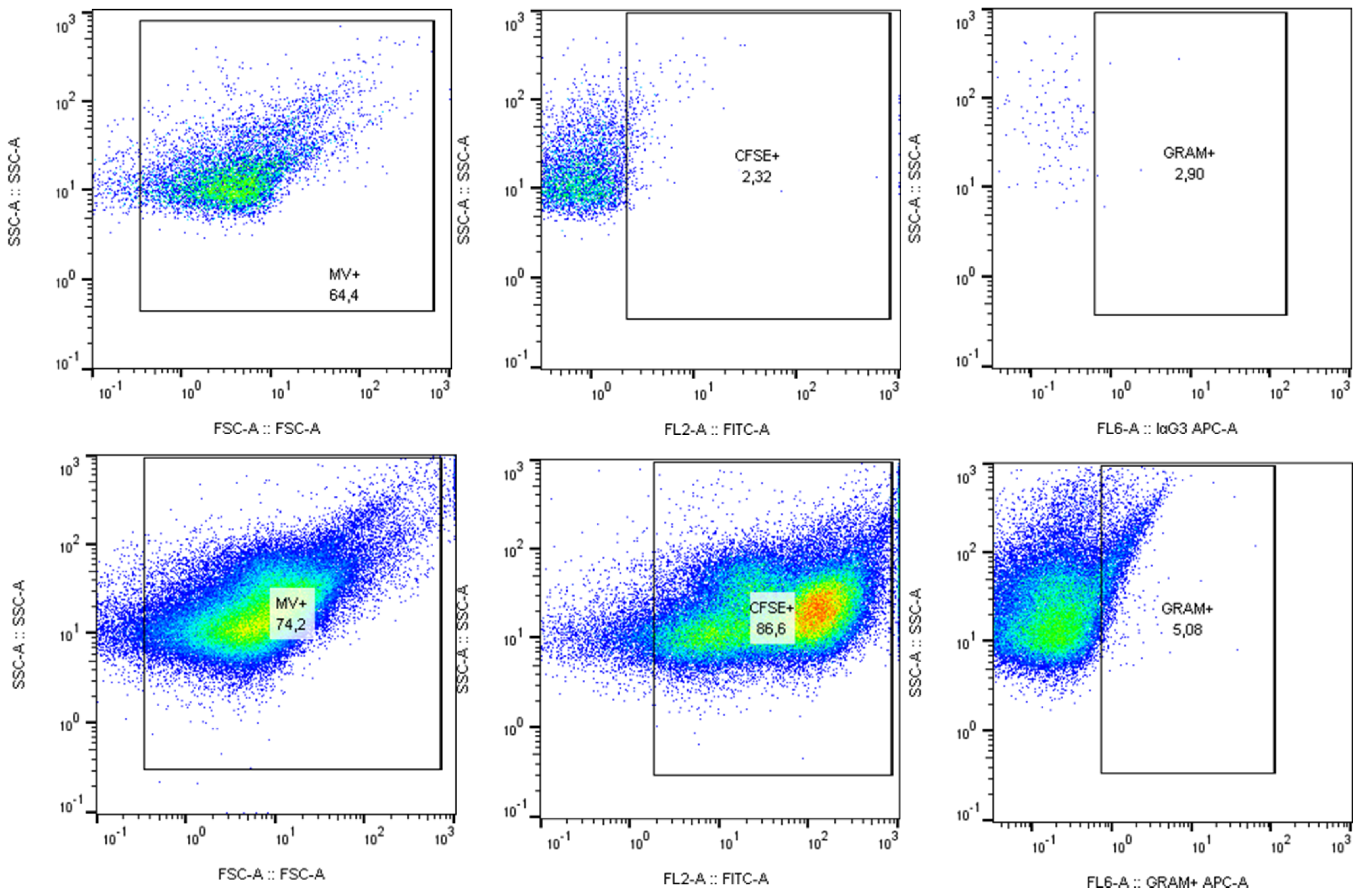

Supplementary Figure S3: CD294+ EVs gating protocols for plasmatic EVs. A-C negative control with PBS; D-F Plasma samples. A, D): scatter plots; B, E): dot plots with gating for CFSE fluorescence; C, F): dot plots for experimental sample (scatter+ CFSE + ANTI- CD294+ antibody); D: dot plots for scatter+ ANTI-CD294+ antibody

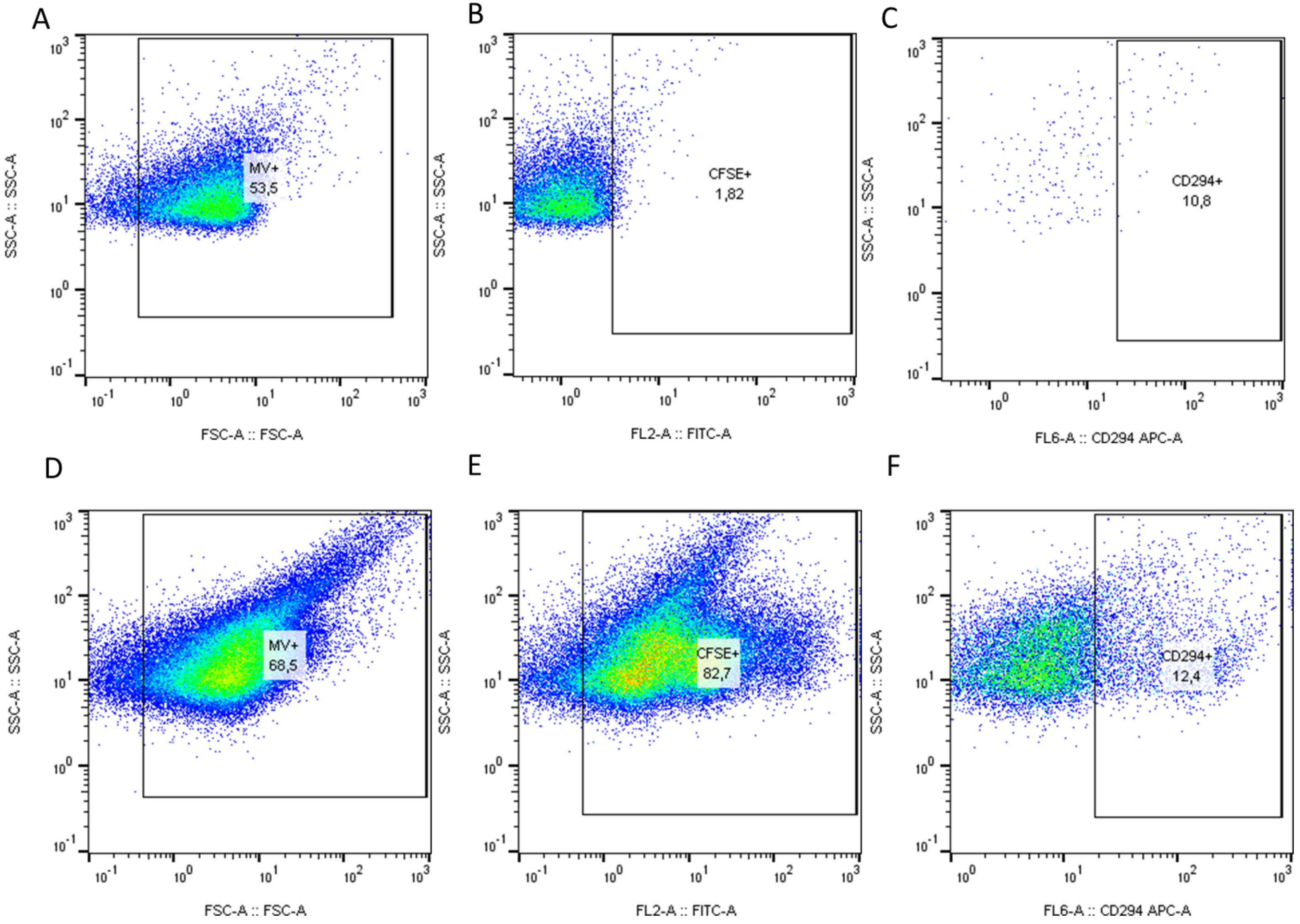

Supplementary Figure S4: CD203+ EVs gating protocols for plasmatic EVs. A-C negative control with PBS; D-F Plasma samples. A, D): scatter plots; B, E): dot plots with gating for CFSE fluorescence; C, F): dot plots for experimental sample (scatter+ CFSE + ANTI- CD03+ antibody); D: dot plots for scatter+ ANTI- CD203+ antibody

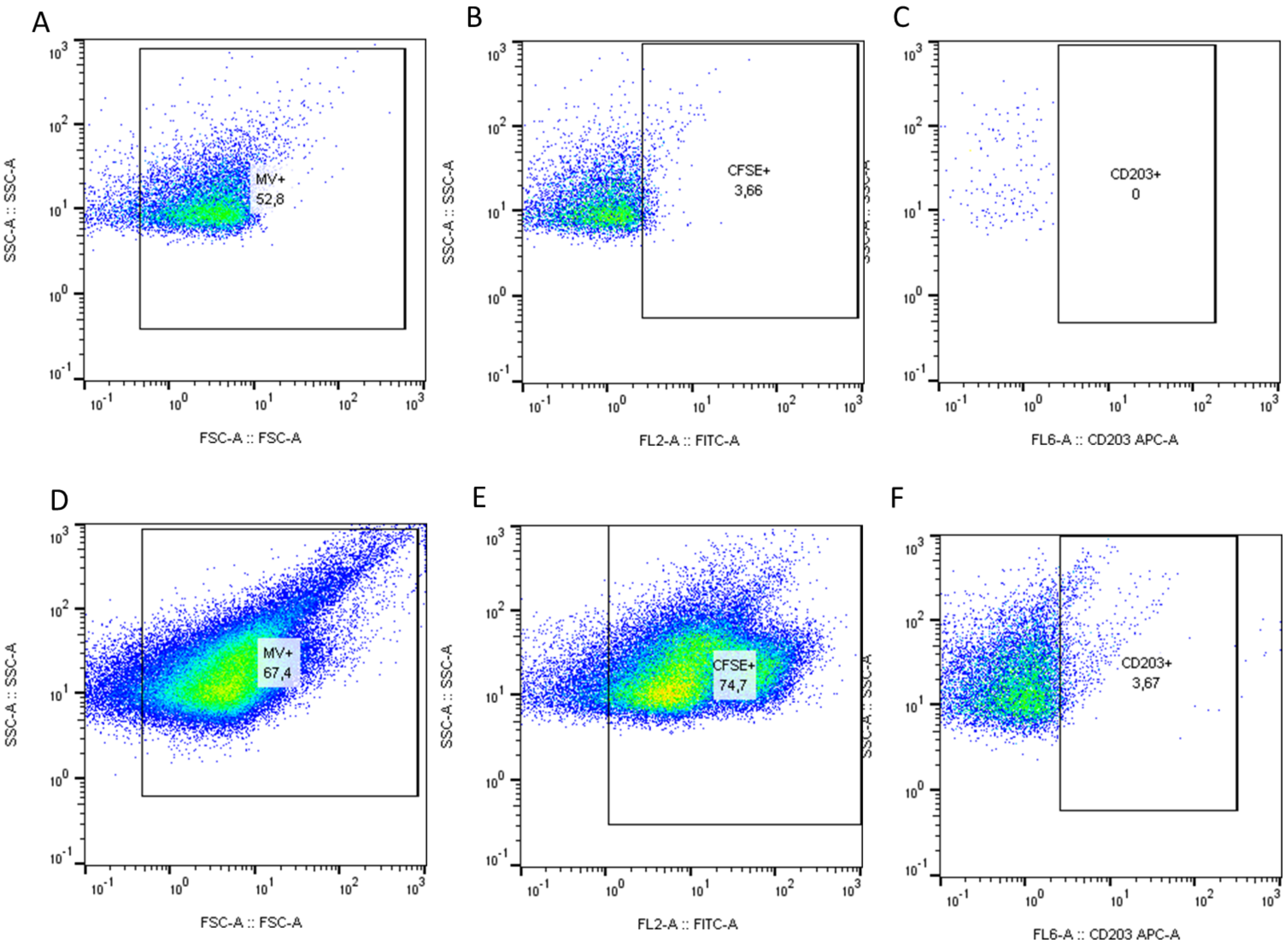

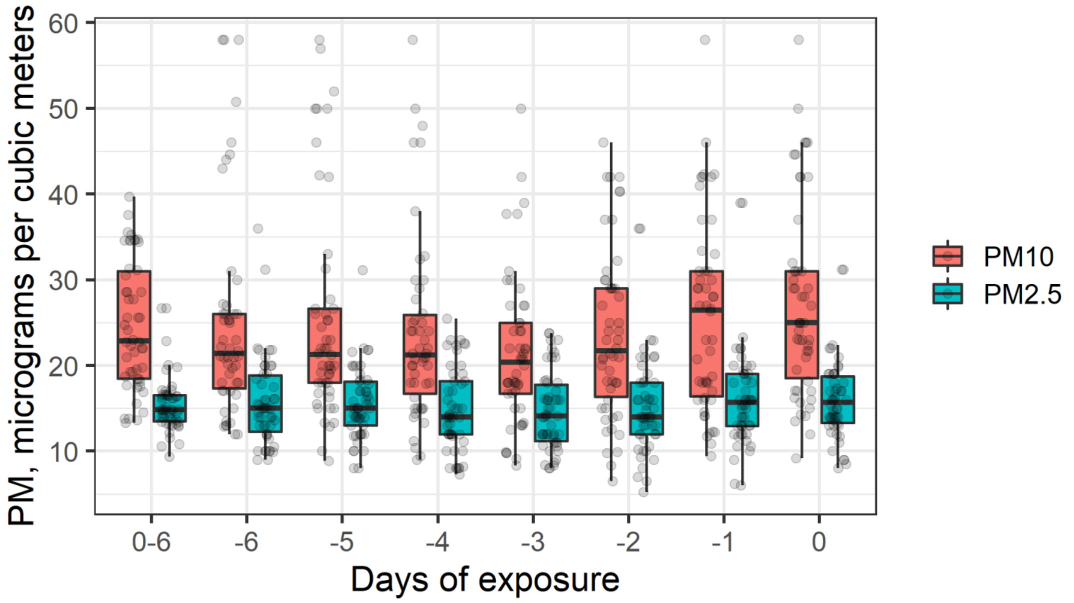

***Supplementary Figure S5. PM10 and PM2.5 Daily exposure levels** from day 0 to day -6 and mean levels (day 0-6).*

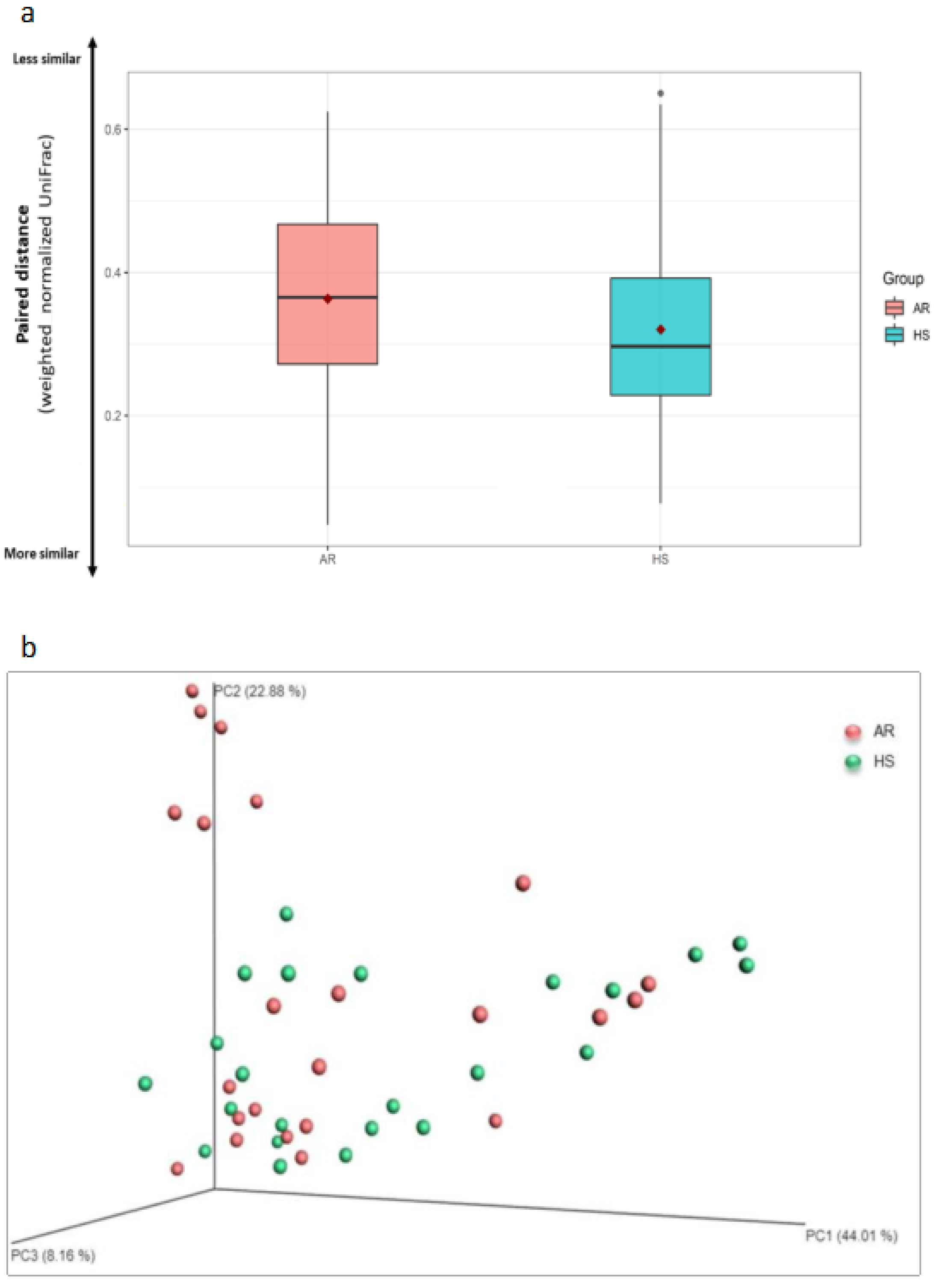

***Supplementary Figure S6. bNM diversity**. a) Intra-subject paired distance of the AR and HS group; b) Principal coordinate analyses (PCoA) plot made using the normalized Weighted UniFrac distance metric, each dot corresponds to a single subject belonging either to AR (red dot) or HS (green dot) groups, the variance explained by each axis is given in parenthesis on bNM composition*
